# Temperature-Dependent Dynamic Nuclear Polarization of Diamond

**DOI:** 10.1021/acs.jpcc.5c02747

**Published:** 2025-06-28

**Authors:** Gevin von Witte, Aaron Himmler, Konstantin Tamarov, Jani O. Moilanen, Matthias Ernst, Sebastian Kozerke

**Affiliations:** † Institute for Biomedical Engineering, 27219 University and ETH Zurich, 8092 Zurich, Switzerland; ‡ Institute of Molecular Physical Science, ETH Zurich, 8093 Zurich, Switzerland; ¶ Department of Technical Physics, 4344 University of Eastern Finland, 70210 Kuopio, Finland; § Department of Chemistry, Nanoscience Center, 4168 University of Jyväskylä, 40014 Jyväskylä, Finland

## Abstract

Dynamic nuclear polarization (DNP) can increase nuclear magnetic resonance signals by several orders of magnitude. We report on ^13^C DNP experiments in diamond at 3.4 and 7 T static magnetic fields in a temperature range of 300 to 1.7 K. Nuclear polarization enhancements between 100 and 600 were measured for all temperatures, corresponding to polarizations between 0.1% (300 K) and 38% (1.7 K) at 7 T. A strong temperature dependence of the DNP profiles was observed with broad lines at low temperatures and more structured features at room temperature. Longitudinal-detected electron paramagnetic resonance (EPR) experiments revealed an additional broad temperature-dependent electron line centered around the *m*_I_ = 0 line of the P1 triplet transitions. This additional electron line leads to an asymmetry of the low-temperature EPR spectrum and might arise from clustered P1 centers or other nitrogen defects in diamond, e.g., N2 or N3 centers, which are known to shorten P1 electronic relaxation times. Our results suggest that nuclei are preferentially polarized via a direct hyperfine mediated polarization transfer, while nuclear spin diffusion in the sample plays a minor role.

## Graphical abstract

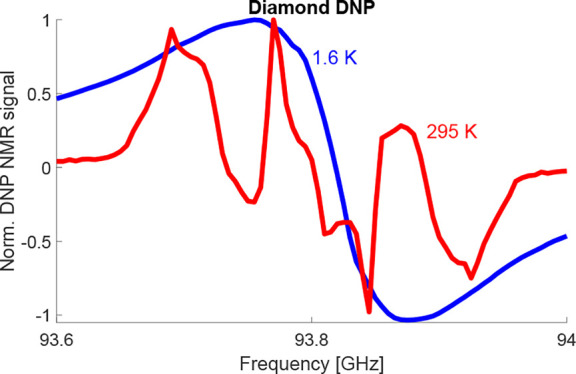

## Introduction

Hyperpolarization of diamond with P1 centers at liquid-helium temperature has been investigated for hyperpolarized nanoparticle magnetic resonance imaging (MRI) applications.^1–4^ Long-lasting nuclear polarizations of a few tens of percent have been achieved at a few Tesla magnetic fields. The P1 DNP profiles around 3.5–4 K revealed two broad DNP lobes for either positive or negative enhancement.^1,3^

Room temperature hyperpolarization of diamond with P1 centers under static^1,5–8^ and magic angle spinning conditions^9^ showed enhancements exceeding 100 at several Tesla magnetic fields. The DNP profiles at room temperature revealed a large number of narrow peaks ascribed to different hyperpolarization mechanisms including solid effect (SE), cross effect (CE) and truncated cross effect (tCE).

In this work, we study the hyperpolarization of ^13^C in diamond by DNP in a static magnetic field of 3.4 and 7 T and a temperature range from 1.6 to 300 K. Nuclear polarization enhancements exceeded a factor of 100 under all conditions and polarization levels of up to 38% were found. The temperature-dependent changes of the DNP profile were complemented by longitudinal-detected (LOD) electron paramagnetic resonance (EPR) experiments. In addition to the three hyperfine-split electron lines of the P1 centers, a temperature-dependent broad electron line was detected. The interplay between the different electron systems and their influence on DNP are discussed.

## Methods

### Sample

High-pressure high-temperature (HPHT) synthesized monocrystalline diamonds with an average particle size of 10 ± 2 μm were purchased from Microdiamant AG (Switzerland). In the Supporting Information, three other diamond samples are characterized using EPR: (i) < 10 nm diamonds from Sigma-Aldrich (USA), (ii) nanodiamonds up to a size of 250 nm from Microdiamant AG (Switzerland) and (iii) microdiamonds with 2 ± 0.5 μm average particle size as previously reported.^10^ All diamonds were used as purchased without further treatment.

### Dynamic Nuclear Polarization

The DNP measurements were performed on home-built polarizers at 3.4 and 7 T^11^ with temperatures ranging from 1.6 to 300 K. The 3.4 T (142 MHz ^1^H Larmor frequency) polarizer was equipped with an OpenCore NMR spectrometer^12–14^ and the 7 T (299 MHz ^1^H Larmor frequency) with a Bruker Avance III console (Bruker BioSpin AG, Switzerland). At 3.4 T, a VDI (Virginia Diodes Inc., USA) microwave source with 400 mW output power was coupled to a stainless steel waveguide. At 7 T, a VDI microwave source with 200 mW output power was connected to an in-house electroplated low-loss, silver-coated stainless steel waveguide.^15^ This permitted a similar microwave power in the sample space (estimated at 65 mW for the 7 T polarizer) for both setups. Other details of the setups are described elsewhere.^11,15^

The absolute values of the polarization were calculated based on the average of two thermal equilibrium measurements (saturation, waiting time of approximately three times the nuclear spin–lattice relaxation time, followed by detection with a large flip angle pulse) at 3.4 K.

### Electron Paramagnetic Resonance

EPR spectra at 3.4 or 7 T were acquired with our in-house developed longitudinal-detection (LOD) EPR setup.^15,16^ For LOD EPR measurements, a different coil and sample holder needed to be mounted on the cryostat insert while the MW setup remained identical. The VDI MW source was controlled through an attenuation voltage from a digital acquisition board (DAQ, National Instruments, USA) fed into the TTL input of the MW source. The EPR signal was detected using a home-built copper coil and the voltage was amplified before detection at 1 MHz sampling rate with the same DAQ.

X-band EPR spectra were measured at room temperature with a Magnettech MiniScope MS5000 (Bruker Corp.). The samples were filled into EPR tubes and placed at a controlled height within the spectrometer cavity which was automatically adjusted. The spectra were taken with 30 dB attenuation at a modulation amplitude of 0.2 mT and a modulation frequency of 100 kHz. The *g*-factor was verified and the number of spins was calculated using a standard TEMPO sample. The uncertainty of measurements was estimated with separate technical replicates (multiple measurements of the same samples) of both TEMPO and a control porous Si sample.^17^

### Data Analysis

All data processing was performed with in-house developed MATLAB (MathWorks Inc., USA) scripts. All measurement uncertainties of the processed experimental data result from the 95% fit intervals unless otherwise stated. Experimental instabilities such as changes in the MW output power or minor temperature fluctuations as well as uncertainties in the thermal equilibrium measurement were considered negligible. We analyzed the NMR data in the time domain, fitted the FID with a combination of three oscillating exponentials (real part of the signal) and used the maximum of the fit (cf. Figures S1 and S2, Supporting Information). Among the possible models to fit the polarization build-ups and decays, we chose a stretched exponential function (cf. Eq S1 and Figures S3 and S4, Supporting Information).

A comparison of the different analysis and fit methods can be found in Figure S2, Supporting Information.

## Results

### Dynamic Nuclear Polarization at Different Temperatures and Fields

Amplification by DNP was efficient with enhancements exceeding 100 at all temperatures between 1.7 and 300 K at 7 T (cf. Figure 1b). The observed room temperature polarization at 7 T of 0.09% (enhancement of 150 relative to thermal ^13^C polarization at 7 T and room temperature) exceeds the achievable room temperature nuclear hyperpolarization with NV centers in diamond microparticles of around 0.04% (enhancement of 1500 at 0.29 T).^18^ Lowering the temperature from 300 to 3.4 K resulted in an approximately exponential increase of the polarization (cf. Figure 1a). Lowering the temperature from 3.4 to 1.7 K increased the electron polarization from 88 to 99% and the nuclear polarization from 32 to 38%. Together with the approximately exponential increase in polarization with decreasing temperature, this suggests that the nuclear hyperpolarization depends only on the thermal electron polarization and not on changes in electronic relaxation properties as often encountered with cooling. Using notions of compartment modeling,^19–21^ the balance between DNP injection and relaxation appears to be independent of temperature such that the increase in ^13^C polarization levels with temperature is only due to increased thermal electron polarization.

**Figure 1:**
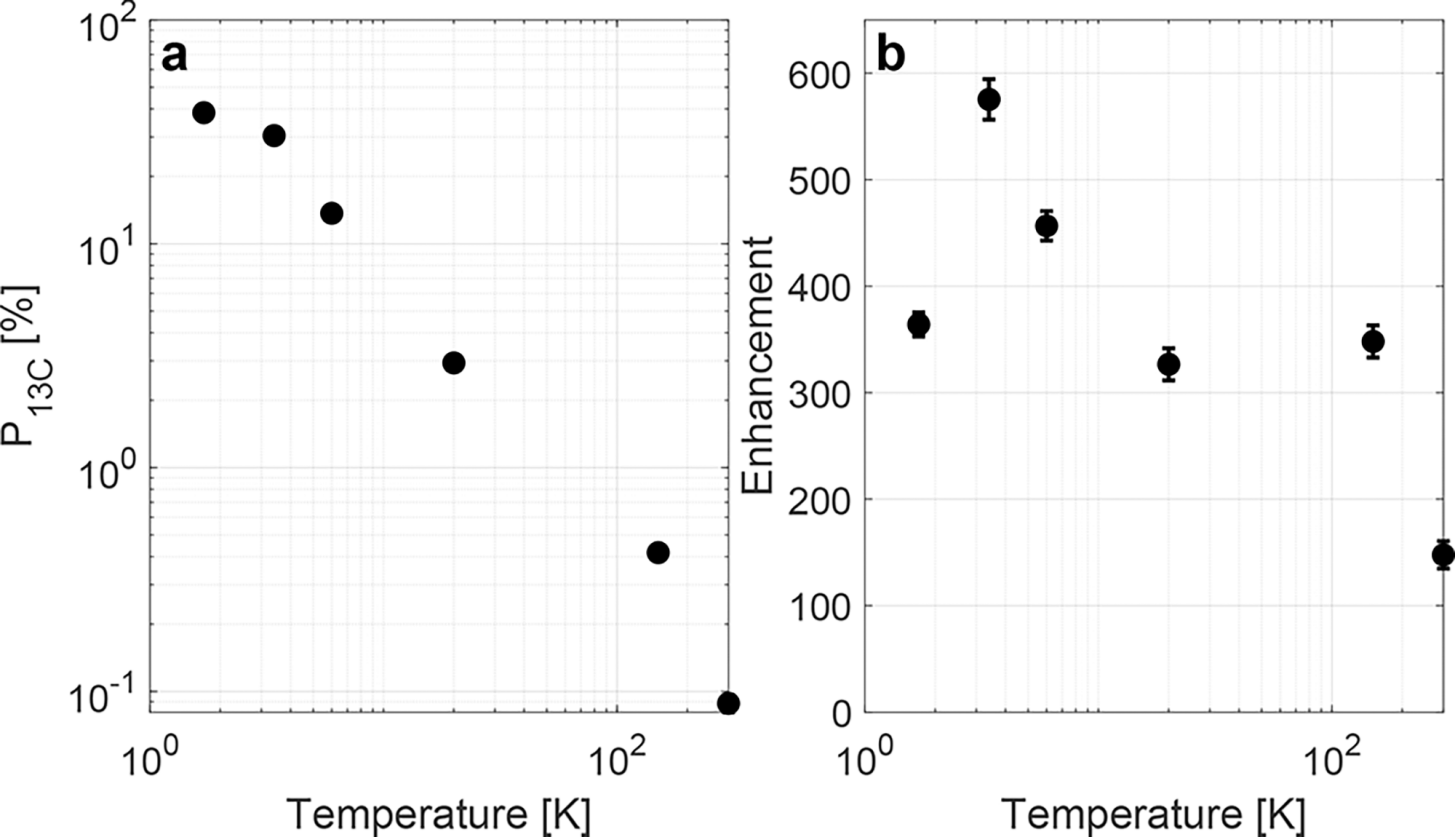
(a) Steady-state nuclear hyperpolarization levels and (b) enhancements between 1.7 and 300 K at 7 T and 196.830 GHz.

Figure 2 compares the DNP profiles between 1.6 and 295 K for 3.4 and 7 T. At room temperature (Figure 2a), several peaks in the DNP profile can be identified which can be linked to solid-effect (SE), cross-effect (CE) and truncated cross-effect (tCE) DNP. Solid-effect DNP relies on MW irradiation at ω_e_ ± ω_n_ with ω_e_ (ω_n_) being the electron (nuclear) Larmor frequencies. The electron-to-nuclear polarization transfer at the two frequencies relies on electron–nuclear state mixing via strong hyperfine couplings. Cross-effect DNP relies on triple spin flips involving two electrons and one nucleus. Triple spin flips are possible, if the energy difference of the two electrons matches the nuclear Larmor frequency (|ω_e1_ – ω_e2_|≈ ω_n_). Hyperpolarization via CE occurs if the two electrons have a polarization difference, which can be achieved through MW irradiation. Typically, CE peaks appear separated by ω_n_. The truncated CE is a special type of CE, with the two electrons having vastly different electronic relaxation times, causing one electron to be nearly saturated by MW irradiation while the other appears unsaturated.^5,7,22^ Enhancement by tCE appears at the frequency of the electron resonance^5^ unless positive and negative tCE enhancements cancel.^7^ In Figure 2, the approximate frequencies of the DNP mechanisms (SE, CE, tCE) are indicated with vertical lines. The different DNP mechanisms (SE, CE, tCE) have different contributions to the observed DNP enhancements, which was discussed in detail for 3.4 ^5^ and 7 T^7^ at room temperature, e.g., SE from the *m*_I_ = ±1 electron lines appears absent at 7 T. The finite width of the electron lines in diamond leads to a finite width of the DNP lines, possibly causing an overlap of contributions from different DNP mechanisms. In addition, the complex *m*_I_ = ±1 electron line shape arises from the powder averaging of the different defect orientations,^5^ possibly leading to differences between the observed DNP peaks and the averaged hyperfine couplings used to indicate the frequencies of the DNP contributions in Figure 2. In this work, we refrain from a detailed discussion of the high-temperature DNP mechanisms and explanations of the DNP profile as these have been discussed before.^5,7^ Instead, we focus on the dependence of the DNP profile and electron spectra on temperature.

**Figure 2:**
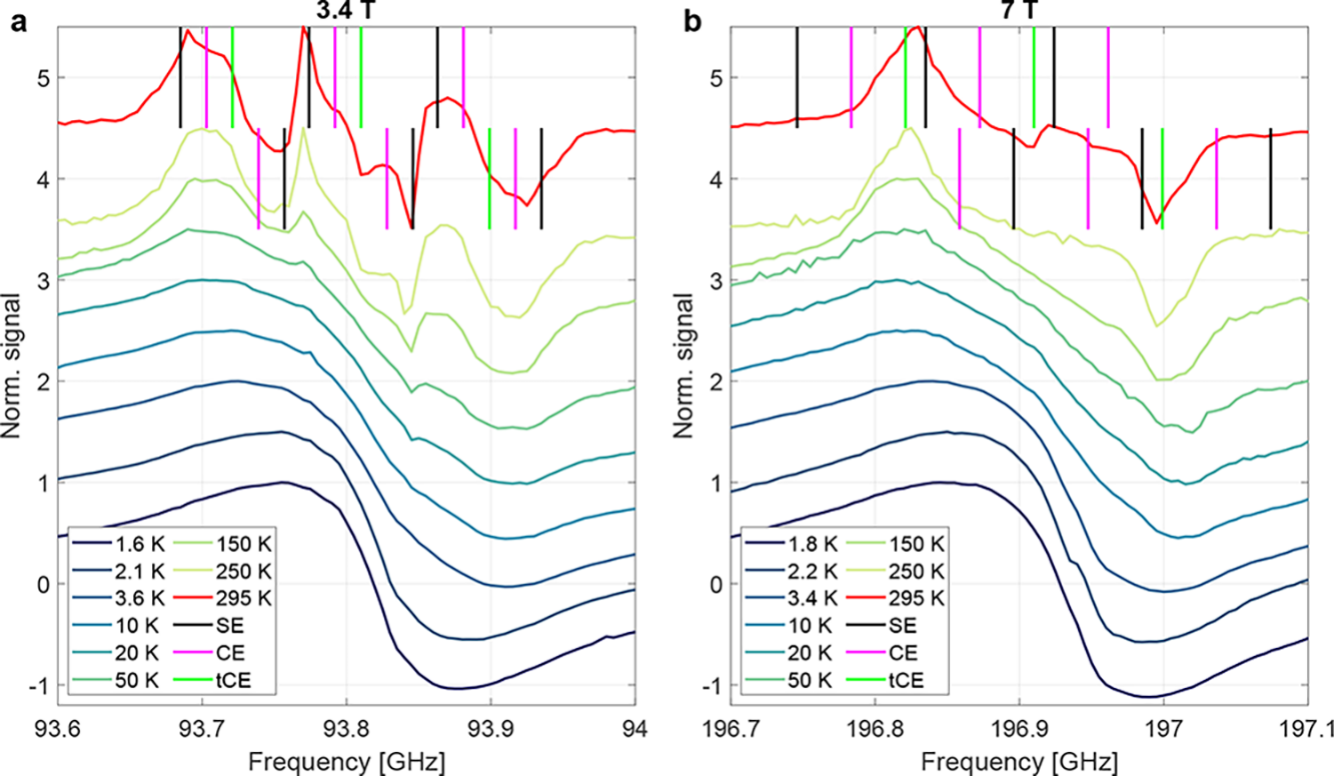
Selected DNP profiles between 295 and 1.6 K for (a) 3.4 T and (b) 7 T with expected frequencies of different DNP mechanisms (SE: solid effect, CE: cross effect, tCE: truncated cross effect) indicated by vertical lines. An extended data set of DNP profiles can be found in Figure S5, Supporting Information. DNP profiles are vertically offset by 0.5 for clarity and twice the offset for 295 K.

At 3.4 T (Figure 2a), most of the distinct features of the high-temperature profile disappear below 50 K. Below 50 K, the DNP profile shows two broad DNP lobes with nearly symmetric intensities for spin-up and spin-down DNP. At 7 T (Figure 2b), the high-temperature profile is dominated by two triangularly shaped peaks. These peaks become smoother with decreasing temperatures. An additional smaller peak at the center of the DNP profile becomes visible at 295 K but becomes difficult to observe for temperatures below 250 K. DNP in diamond at cryogenic temperatures is discussed in more detail in Section S2, Supporting Information, owing to the incomplete understanding of the electron spin systems involved in the DNP process.

### Electron Spin System

To better understand the observed changes in the DNP profiles of diamond, we performed longitudinal-detected (LOD) EPR measurements under DNP conditions (see Methods). Due to improvements in our LOD EPR setup,^15^ we were able to detect the diamond LOD EPR signal from 3.3 to 300 K. As evident in the EPR spectra shown in Figure 3a–c, the ^14^N hyperfine coupling to the P1 center is around 92 MHz, which is in agreement with the literature values of *A*_∥_ = 82 MHz and *A*_⊥_ = 114 MHz.^23^ However, the observed LOD EPR spectra cannot be explained based on three peaks originating from the ^14^N hyperfine split P1 electron system alone. To fit the observed LOD EPR spectra, a combination of a Lorentzian line for the ^14^N *m*_I_ = 0, two Gaussians for the *m*_I_ = ±1 and an additional broader Gaussian line was assumed. Specifically, we used

$$\begin{array}{cl} S_{\mathrm{EPR}} & =\frac{S_{P1}}{\sqrt{2\pi }\sigma_{\pm 1}}[e^{-(\nu -(\nu_{P1}-A_{P1}){)}^{2}/2\sigma_{\pm 1}^{2}}+e^{-(\nu -(\nu_{P1}+A_{P1}){)}^{2}/2\sigma_{\pm 1}^{2}}]\cdot \cdot \cdot \\ & +\frac{S_{P1}}{\pi }\frac{\sigma_{0}}{(\nu -\nu_{P1}{)}^{2}+\sigma_{0}^{2}}+\frac{S_{b}}{\sqrt{2\pi }\sigma_{b}}e^{(\nu -\nu_{b}{)}^{2}/2\sigma_{b}^{2}}+S_{\mathrm{offset}} \end{array}$$ 1

where 0 and ±1 subscripts indicate the different ^14^N hyperfine contributions of the P1 centers; the b subscript refers to a broad Gaussian line; σ are the respective line widths; *S* are the signal amplitudes of the different contributions; ν_P1_ refers to the P1 center frequency of the *m*_I_ = 0 contribution and *A*_P1_ is the orientation averaged hyperfine coupling of the *m*_I_ = ±1 contributions. The assumed model ensures that all ^14^N hyperfine contributions have the same intensity (area under the curve (AUC), total number of electrons) and the Gaussian line shape for the *m*_I_ = ±1 contributions approximates the powder broadening^5^ of these lines due to the hyperfine coupling. An example of the quality of the fit of the model applied to a measured EPR spectrum is shown in Figure 3a–c for LOD profiles at 3.3, 35, and 295 K.

**Figure 3:**
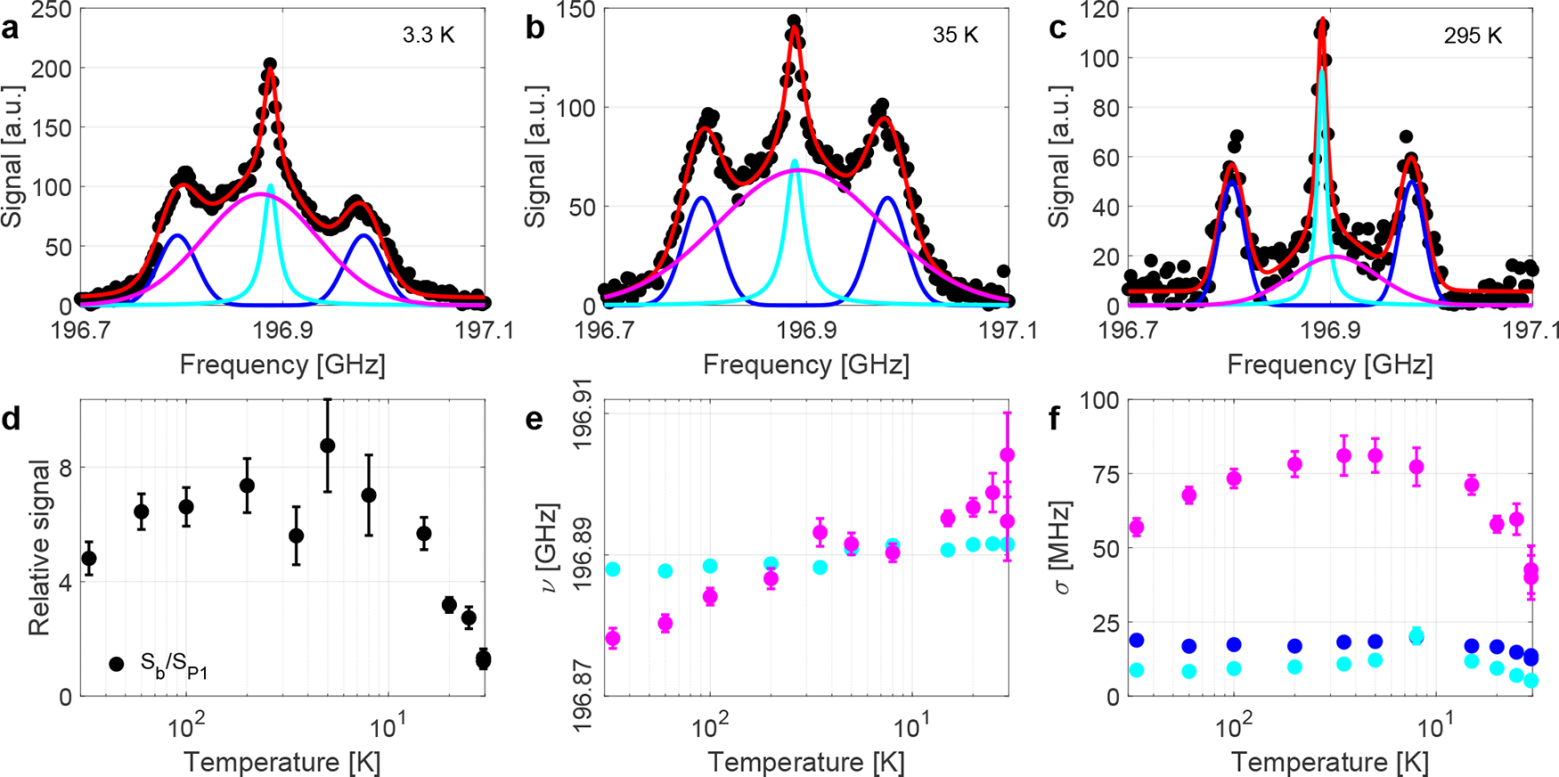
(a–c) Longitudinal-detected (LOD) electron paramagnetic resonance (EPR) profiles at 7 T for different temperatures. The LOD EPR spectra are fitted with eq 1 as described in the main text. (d) Comparison of the signal amplitude of the broad component compared to the ^14^N hyperfine-split P1 center line. (e) Fitted center frequencies of P1 centers (*m*_I_ = 0, cyan) and the broad (magenta) component. The broad component has a similar *g*-factor as the P1 center although a weak temperature dependence. (f) Line widths of the Gaussian *m*_I_ = ±1 (blue), Lorentzian *m*_I_ = 0 (cyan) and broad Gaussian (magenta) lines. Uncertainties can be smaller than the symbols for all fit parameters. All data was acquired at 7 T and with full MW power. Lower MW power causes a reduced signal (cf. Figure S11, Supporting Information) but results in qualitatively similar LOD profiles (data not shown but available, cf. Data Availability section).

The fits reveal a large contribution of the broad component to the total LOD EPR signal at low and intermediate temperatures as shown in Figure 3d. At 300 K, the LOD EPR profile has only a weak broad component. This could be due to a low detection efficiency of the LOD EPR due to insufficient saturation for lines with short relaxation times. The resonance frequency of the broad component as given by its *g*-factor is similar to the resonance frequency of the P1 center. In contrast to the P1 center, the center frequency of the broad component appears weakly temperature dependent relative to the frequency of the P1 center (cf. Figure 3e), which rationalizes the observed asymmetry of the EPR spectra at low temperature. Moreover, the line width of the broad component shows a more pronounced temperature dependence (cf. Figure 3f) with the largest line widths at intermediate temperatures of tens of kelvin and the narrowest line at 300 K.

### Microwave Frequency and Power

Figure 4a compares nuclear hyperpolarization build-up curves at 3.5 K and 3.4 T with curves at 3.4 K and 7 T for two different microwave frequencies (highest DNP enhancement at 196.83 GHz with lower DNP enhancement at 196.5 GHz, cf. Figure 2b). While measurements at 7 T in general show a slower polarization build-up, the polarization build-up appears to be independent of the MW frequency.

**Figure 4:**
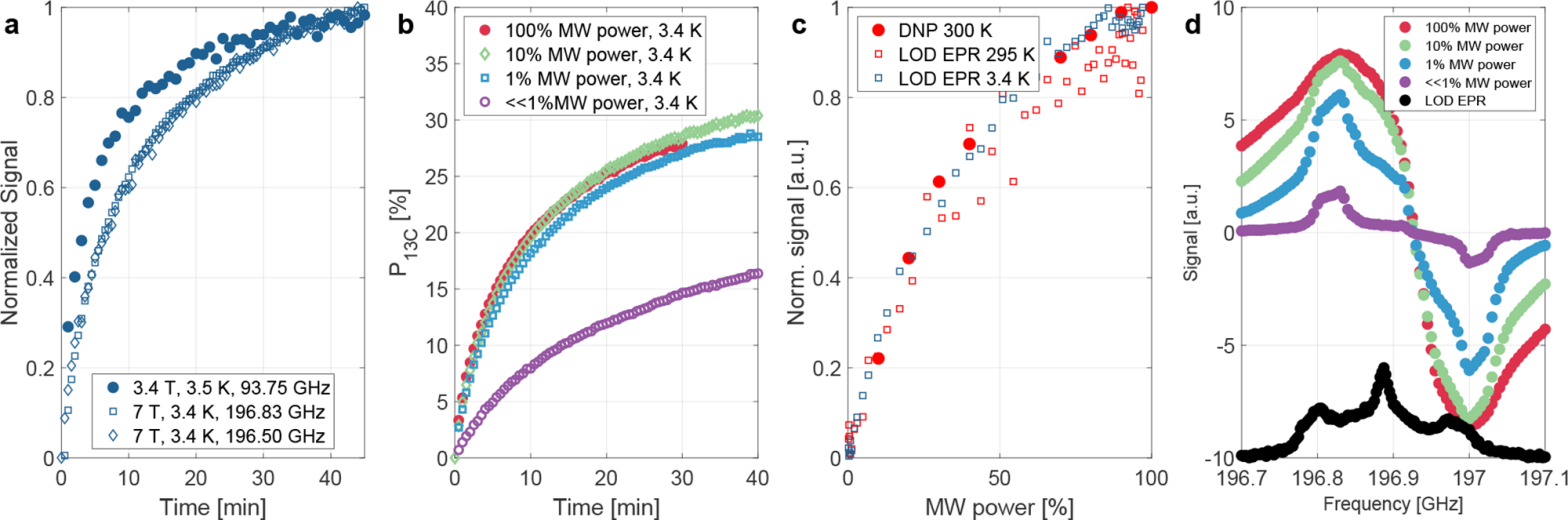
(a) Hyperpolarization build-up experiments around 3.4 K for 3.4 and 7 T. At 7 T, the build-up dynamics is independent of the MW frequency and in general slower than at 3.4 T. At 7 T, 3.4 K and 196.50 GHz, a nuclear polarization of 5.8 ± 0.2% is reached compared to 31 ± 2% at 196.83 GHz. (b) Power dependence of the build-up at 3.4 K, 7 T and 196.83 GHz. For the lowest power, the power is not exactly known but far below 1% of the maximum MW power available. The fit parameters can be found in Figure S4, Supporting Information. (c) Power dependence of the DNP signal after 60 s at 196.830 GHz (7 T) and 300 K (filled symbols) and of the LOD EPR signal at 3.4 and 295 K (open symbols). Each data set is normalized to its respective maximum measured signal. LOD EPR power curves between 3.4 and 295 K are analyzed in Section S3 and in Figure S11 of the Supporting Information. (d) DNP profiles for different MW output powers overlaid with the LOD EPR spectrum at 3.4 K and 7 T with 15 s of DNP prior to detection.

Figure 4b,c compare the power dependence of the DNP signal at 7 T for 3.4 and 300 K. At 300 K, the DNP signal shows a strong dependence on the MW power, with the signal (cf. Figure 4c) increasing by more than a factor of 4, if the MW power increases from 10 to 100% (approximately 20 to 200 mW output power and 6.5 to 65 mW at the sample space, cf. Methods). The nearly identical DNP build-up curves for 1, 10 and 100% MW power at 3.4 K (cf. Figure 4b) are in contrast to the pronounced power dependence of the LOD EPR spectra as displayed in Figure 4c. At 295 K, the LOD EPR and DNP signal both directly follow the saturation of the electron line (cf. Section S3, Supporting Information). At 3.4 K, the LOD EPR signal follows a similar trend as at 295 K, while the DNP signal appears independent of the MW power for MW powers larger than 1% and, therefore, independent of the electron saturation (cf. Figure 4c). With MW powers much lower than 1%, the nuclear steady-state polarization decreases and the build-up time increases (cf. Figures 4b and S4 Supporting Information). We emphasize that it is possible to achieve a nuclear hyperpolarization of 20% for MW powers at the sample much lower than 1 mW.

Reducing the MW power changes the shape of the DNP profile (cf. Figure 4d). This is more prominent at MW powers below 1% than at MW power of 10%, with narrow peaks at 196.83 and 197.00 GHz combined with wider shoulders around the electron resonance frequency. The frequency of 196.83 GHz coincides with the frequencies of the highest DNP enhancements for higher MW powers and is identical to the maximum at 295 K. The 30–40 MHz shift between the LOD and DNP profiles results from the temperature- and impurity-dependent diamagnetic susceptibility of the copper^24,25^ LOD EPR Helmholtz coil.^15^

## Discussion

The room temperature DNP profiles at 3.4 and 7 T shown in Figure 2 are similar to those presented in refs 5 and 7. Nitrogen concentrations between 10 and 100 ppm, 110–130 ppm or less than 200 ppm were reported for the samples used in refs 5 and 7, while our sample contained around 54 ppm of defects of which around 58% are P1 centers (cf. Section S4, Supporting Information). This suggests that the change of DNP and LOD EPR measurements with temperature reported herein appear representative for other diamond microparticles too.

We emphasize that it is challenging to understand the DNP in diamond owing to a range of different defects, the interplay between these, possible spatial inhomogeneity in terms of defect distribution, and different sample manufacturing procedures. In the following, we will discuss the observed temperature-dependent changes of DNP in diamond.

### Polarization Pathway

The 10 ± 2 μm-sized diamond sample studied contained around 54 ppm of defects of which 58% were P1 centers (cf. Section S4, Supporting Information). Assuming a homogeneous distribution of defects throughout the sample’s bulk, the average distance between two unpaired electrons is estimated as *r*_e–e_ ≈ *n*_e_^–1/3^ ≈ 4.7 nm with *n*_e_ the electron concentration per unit volume. The dipolar hyperfine coupling prefactor is *d*_hfs_ = μ_0_/8π^2^ · *ℏ*γ_e_γ_^13^C_/(*r*_e–e_/2)^3^ ≈ 1.5 kHz for a nuclear spin at *r*_e–e_/2 away from an electron (we ignore the other neighboring electrons for simplicity). The *zz*-part of the hyperfine coupling describing the energetic shift of a nuclear spin has an additional prefactor of 2 and an angular dependence ((3cos^2^θ – 1)/2 with θ denoting the angle between the two spins and the main magnetic field *B*_0_). Therefore, most nuclear spins will have a hyperfine coupling of a few kHz to an electron spin in their vicinity.

We note that the hyperfine coupling exceeds the nuclear dipolar zero-quantum (ZQ) line width Δν_ZQ_ as simulated from first principles (Δν_ZQ_ ≈ 200 – 400 Hz, which corresponds to a nuclear spin diffusion coefficient of 20–40 nm^2^/s in a lattice approach or 4–8 nm^2^/s in a nearest neighbor approach^26^). However, for spins with energy differences exceeding the ZQ line width, the probability of nuclear dipolar flip-flops, which are macroscopically considered as nuclear spin diffusion, vanishes. Hence, nuclear spin diffusion is suppressed in the sample. Owing to the small average nearest neighbor electronic and nuclear dipolar couplings of 0.5 MHz and 100 Hz, electron–nuclear four-spin flip-flops^21^ do not lead to a significant nuclear spin diffusion either. The absence of nuclear spin diffusion in diamond is in agreement with findings in ref 27. The combination of few kHz hyperfine couplings and suppressed nuclear spin diffusion suggests that nuclei are preferentially hyperpolarized by a direct electron–nuclear polarization transfer.

We note that the above estimate for the ZQ line width of 200–400 Hz, which is similar to the experimentally accessible single-quantum (SQ) line width,^26^ is in good agreement with measured line widths in low defect diamonds of around 250 Hz.^28^ Moreover, the nuclear line widths are comparable to natural abundance silicon particles despite the lower natural abundance of ^13^C (4.7% ^29^Si vs 1.1% ^13^C natural abundance), which is compensated by the smaller lattice constant of diamond (3.567 Å for diamond compared to 5.431 Å for silicon) and the larger gyromagnetic ratio of ^13^C. Similar to silicon,^26^ the estimated spin diffusion coefficient in diamond would only need seconds to cover distances of several nanometers, which is much faster than the hyperpolarization build-up and decay times exceeding ten minutes (cf. Section S1, Supporting Information). Therefore, if spin diffusion would be present and be much faster than the observed experimental time scales, a monoexponential polarization dynamics similar to ^1^H in glassy matrices could be expected with a single-compartment rate equation model.^19,20^ However, we find a stretched exponential polarization dynamics as discussed in the following.

Further evidence for a limited role of nuclear spin diffusion and a larger influence of direct hyperpolarization from electrons to nuclei comes from the stretched exponent of the fitted build-up curves (cf. Section S1, Figures S3b and S4c of the Supporting Information) with most of the exponents being around 0.8. In Section S6, Supporting Information, a rate-equation model of hyperpolarization for infinitely many uncoupled compartments (without spin diffusion, only hyperpolarization and relaxation by hyperfine coupling to the central electron with *r* ^–3^ scaling) is discussed. The case of infinitely many uncoupled compartments describes the long-time behavior of systems without spin diffusion and only direct DNP and relaxation through the electrons. For short time scales, systems with paramagnetic relaxation and without nuclear spin diffusion have been described with a stretched exponent of 0.5,^29,30^ while we find exponents close to 2/3 (cf. Section S6, Supporting Information). For hyperpolarization with fast spin diffusion compared to the hyperpolarization injection (*k*_W_) and relaxation (*k*_R_) rate constants such that the build-up time constant is given by τ_bup_ = (*k*_W_ + *k*_R_)^−1^, a monoexponential build-up is found.^19^ Hence, the stretch exponent of around 0.8 might be interpreted as direct hyperpolarization being the main polarization pathway while spin diffusion plays a minor role.

### Electronic Spin System

The LOD EPR profiles were fitted with a combination of powder broadened P1 centers and a broad (spin-1/2) defect (cf. Figure 3, eq 1 and Section S3, Supporting Information). Inspired by refs 7–9 , the measured LOD EPR profiles were fitted with a combination of narrow and broad P1 centers. The narrower of the two P1 populations is supposed to describe rather isolated P1 centers, while the second broader P1 population is associated with cluster-broadened P1 centers. The fit function for this is

$$\begin{array}{cl} S_{\mathrm{EPR}} & =\frac{S_{P1,n}}{\sqrt{2\pi }\sigma_{\pm 1,n}}[e^{-(\nu -(\nu_{P1}-A_{P1}){)}^{2}/2\sigma_{\pm 1,n}^{2}}+e^{-(\nu -(\nu_{P1}+A_{P1}){)}^{2}/2\sigma_{\pm 1,n}^{2}}]\\ & +\frac{S_{P1,n}}{\pi }\frac{\sigma_{0,n}}{(\nu -\nu_{P1}{)}^{2}+\sigma_{0,n}^{2}}\\ & +\frac{S_{P1,b}}{\sqrt{2\pi }\sigma_{\pm 1,b}}[e^{-(\nu -(\nu_{P1}-A_{P1}){)}^{2}/2\sigma_{\pm 1,b}^{2}}+e^{-(\nu -(\nu_{P1}+A_{P1}){)}^{2}/2\sigma_{\pm 1,b}^{2}}]\\ & +\frac{S_{P1,b}}{\pi }\frac{\sigma_{0,b}}{(\nu -\nu_{P1}{)}^{2}+\sigma_{0,b}^{2}}+S_{\mathrm{offset}} \end{array}$$ 2

with the n and b subscripts referring to narrow (isolated) and broad (clustered) P1 contributions. The central P1 frequency (*g*-factor) and hyperfine coupling was assumed to be identical for the two P1 populations. The fits of eq 2 to the 7 T LOD EPR spectra at 3.3 and 295 K are shown in Figure 5 with the remaining LOD EPR fits shown in Figure S8, Supporting Information, and the fit parameters summarized in Figure S9, Supporting Information. At high temperatures, this two P1 population fitting model works quite well although it is rather insensitive to several fit parameters, e.g. relative signal between narrow and broad components and some of the broadening as displayed in Figure S9b,d, Supporting Information. At temperatures below around 200 K, as exemplified by Figure 5a for 3.3 K, the two P1 population model struggles with fitting the peak frequencies (hyperfine coupling), peak heights, flanks and asymmetry of the LOD EPR profiles.

**Figure 5:**
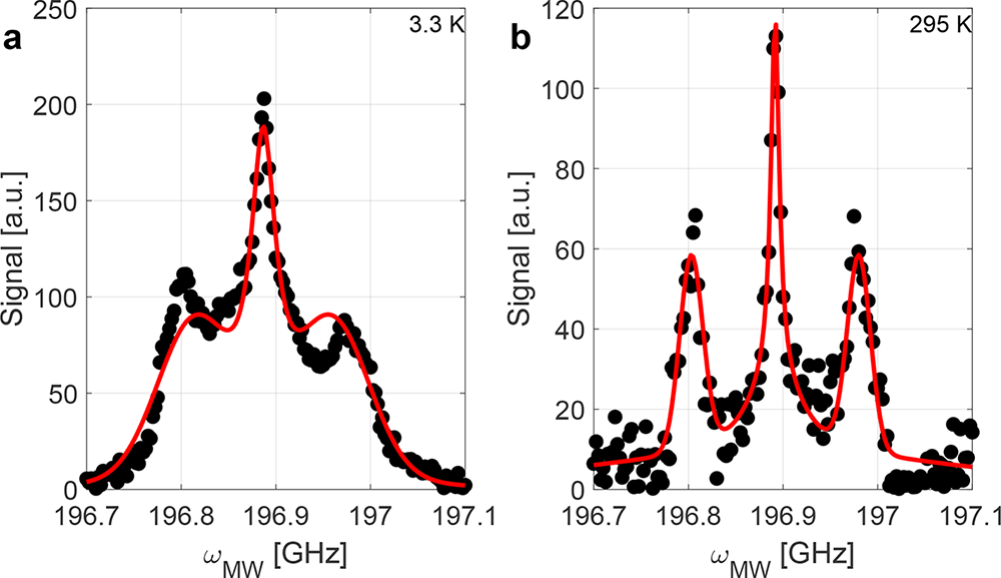
Longitudinal-detected (LOD) electron paramagnetic resonance (EPR) profiles at 7 T and (a) 3.3 K or (b) 295 K. The LOD EPR spectra are fitted with eq 2 as described in the main text.

Fundamentally, a model using a broadened spin-1/2 P1 centers appears not capable of describing the asymmetry observed in the LOD EPR profiles as evident through asymmetric heights between the *m*_I_ = −1 and *m*_I_ = +1 lines - maybe most pronounced at 3.3 K (cf. Figure 5a). If the coupling between the defects would be strong enough to render the clusters effectively as *S* ≥ 1 spin, a population difference between the different spin states might lead to asymmetric lines. The asymmetric electron line could also arise from changes in the MW power reaching the sample at different frequencies and, therefore, the asymmetry might only be an artifact of the measurement. However, the LOD EPR profiles appear symmetric around 50 K (cf. Figure S7, Supporting Information), which indicates that the MW power output is symmetric even at cryogenic temperatures. With the MW source at room temperature, an eventual temperature-dependent change in the MW power reaching the sample would need to arise from the waveguide parts in the liquid helium.

Therefore, the observed asymmetry between the *m*_I_ = −1 and *m*_I_ = +1 lines is most likely an actual feature of the electron spin system. To describe this, we propose a fit model based on a single set of P1 centers and a broad (spin-1/2) defect which describes our sample better (cf. Figures 3, S7, Supporting Information, and eq 1). Owing to the large particle size of 10 ± 2 μm with its low surface-to-volume ratio compared to nanoparticles, it is unlikely that the additional broad line, which contributes around 42% of the total defects in the sample (cf. Section S4, Supporting Information), arises from surface dangling bonds but is considered an additional bulk defect. Different defects have been reported in diamonds with a larger number of nitrogen-based defects, which have similar *g*-factors as the P1 center.^31^ In Section S5, Supporting Information, a selected group of nitrogen bulk defects is discussed. We highlight here that so-called N2 and N3 centers, whose electron lines overlap with the *m*_I_ = 0 line of the P1 center,^32–34^ could explain the observed broad line and the likely shortening of P1 electronic relaxation times as discussed in the next section.

### Electronic and Nuclear Relaxation Times

The total electron concentration of 54 ppm corresponds to around 200 ^13^C nuclei per electron for ^13^C at 1.1% natural abundance in diamond. Considering only the P1 centers, our sample contained around 350 ^13^C nuclei per P1 center.

In ref 35, diamond samples with 25 or 95 ppm of P1 centers and without other (nitrogen) defects were investigated. Accordingly, electronic *T*_1,e_ relaxation times of tens to hundreds of seconds around 10 K with increasing relaxation times upon decreasing temperatures were found. For such long electronic relaxation times, it is difficult to envision how the stretched DNP build-up times of 12 min (cf. Figure S3a, Supporting Information) with up to 38% nuclear polarization at liquid-helium temperatures are feasible from such slow relaxing P1 centers.

References 35 and 36 report samples containing N2 and N3 centers and a shortening of the P1 and nuclear relaxation times was found, suggesting an interplay between the different bulk spin defects. Specifically, measurements at 4.7 T (200 MHz ^1^H Larmor frequency, 50 MHz ^13^C Larmor frequency) and room temperature^34,36^ suggest that a combination of P1 and P2 centers is inefficient in relaxing nuclear polarization even at concentrations of around 5 ppm (*T*_1,n_ > 10 h), while a mixture of P1 centers and N2 (N3) centers with 0.04 (10) ppm leads to *T*_1,n_ around 5.4 (1.4) h. Shortened electron relaxation times of the P1 centers appear consistent with cryogenic X- and Q-band EPR experiments of diamond micro- and nanoparticles designed for hyperpolarized DNP.^1,4,37^ Further evidence for the shortening of P1 electron relaxation times comes from our LOD experiments with LOD time constants of a few hundred μs (cf. Section S3 and specifically Figure S10, Supporting Information). The measured LOD time constants are comparable to other DNP radicals or defects.^15^

Based on these considerations, the broad line detected upon cooling in our LOD EPR experiments could be due to N2 or N3 centers, although other defects cannot be completely ruled out. Both defects might explain a broad line around the *m*_I_ = 0 P1 EPR line, a fast relaxation at room temperature and possibly at low temperatures (subsecond time scale).

### Microwave Power Dependence

At 3.4 K and 7 T, the DNP build-up curves at the frequency with the highest enhancements were independent of MW powers exceeding 1% (cf. Figure 4b), while the LOD EPR signal under the same conditions showed a power dependence (cf. Figures 4c and S11, Supporting Information). In contrast, at 300 K the DNP signal followed the electron saturation as measured in the LOD EPR power curve (cf. Figure 4c).

For TEMPO in ^1^H glassy matrices^20^ at liquid-helium temperatures, a nearly MW power-independent DNP signal was accompanied by a decrease in build-up time for high MW powers. This was attributed to an increase in triple spin flips as in cross effect (CE) DNP, which causes, on the one hand, DNP and, on the other hand, paramagnetic relaxation. However, in the current case, the build-up times for the 1, 10 and 100% MW power measurements were nearly identical (cf. Figures 4b and S4b of the Supporting Information), suggesting a different origin of the MW power-independent signal and with that a possible lack of relevance of triple spin flips.

Far below 1% MW power (MW source not calibrated in this regime), the achievable steady-state polarization decreases and the build-up time is prolonged. For such low MW powers, the rather efficient DNP might be due to isolated defects with very long electronic relaxation times such that even a weak MW field causes electron saturation and enables an efficient DNP creation. This qualitative difference in DNP generation is supported by the changes in the DNP profile with MW power (cf. Figure 4d). The DNP profiles seem to consist of a broad and narrow component. The broad component is dependent on the MW power, while the narrow component is mostly independent of MW power. This could be explained by two different DNP processes with the broad component depending on the broad electron line, while the narrow component depends on the *m*_I_ = ±1 P1 electron lines. The narrow components resemble the shape of powder broadened *m*_I_ = ±1 P1 electron lines (cf. Figure S15, Supporting Information and ref 5).

## Conclusions

In μm-sized diamonds, DNP enhancements of several hundreds between 1.7 and 300 K and at a few Tesla magnetic field are achievable with ≤200 mW of microwave power. When lowering the temperature, the DNP profiles change from feature-rich to broad DNP lobes (positive and negative enhancements), indicative of different DNP origins. Our results suggest that P1 centers and a broad spin-1/2 electron line, tentatively associated with N2 or N3 centers, cause the observed DNP with nuclei hyperpolarized directly via hyperfine coupling rather than through suppressed nuclear spin diffusion. The second type of defect, besides the P1 centers, appears essential to provide electronic relaxation times compatible with DNP build-up and relaxation times. The interplay between different temperature-dependent electron systems may offer new possibilities to study dynamic nuclear polarization.

## Supplementary Material



## Data Availability

The raw experimental data and the Matlab scripts for processing can be found under 10.3929/ethz-b-000709870.

## Notes

Additional information about DNP build-ups, decays, and profiles at different temperatures and fields; LOD EPR at 7 T and X-band EPR of different diamond samples; discussion of the possible defects in diamond; and DNP rate-equation model of infinitely many uncoupled compartments (PDF)
