# Supplementary material for: Temperature-Dependent Dynamic Nuclear Polarization of Diamond
Source: J Phys Chem C Nanomater Interfaces. 2025 Jun 28;129(27):12577–84. doi: 10.1021/acs.jpcc.5c02747 (PMC12257587; doi:10.1021/acs.jpcc.5c02747)
Supplement: Supplementary file 1 [file jp5c02747_si_001.pdf]

Supporting Information for Publication: Temperature-dependent dynamic nuclear polarization of diamond

Gevin von Witte,[†,‡] Aaron Himmler,[‡] Konstantin Tamarov,[¶] Jani O. Moilanen,[§] Matthias Ernst,[‡] and Sebastian Kozerke[*,†]

†*Institute for Biomedical Engineering, University and ETH Zurich, 8092 Zurich, Switzerland*

‡*Institute of Molecular Physical Science, ETH Zurich, 8093 Zurich, Switzerland*

¶*Department of Technical Physics, University of Eastern Finland, 70210 Kuopio, Finland*

§*Department of Chemistry, Nanoscience Center, University of Jyväskylä, 40014 Jyväskylä, Finland*

E-mail: kozerke@biomed.ee.ethz.ch

Contents

S1 Build-ups and decay **S3**

S2 DNP profiles and DNP at cryogenic temperatures **S8**

S3 Longitudinal-detected (LOD) electron paramagnetic resonance (EPR) **S11**

S3.1 LOD profiles at 7 T of 10 µm diamonds S11

S3.2 LOD decay times at 7 T of 10 µm diamonds S13

S3.3 Power dependence at 7 T of 10 µm diamonds S14

S3.4 LOD profiles of nanodiamonds S16

S4 X-band EPR **S19**

S5 Discussion of possible defects **S21**

S6 Uncoupled compartments model of DNP **S24**

References **S28**

S1 Build-ups and decay

The measured free induction decays (FIDs) can be fitted either in the time domain or Fourier-transformed frequency domain as shown in Fig. S1. In the frequency domain, a combination of three pseudo-Voigt functions is used to fit the spectrum. Subsequently, the signal for a given measurement can be evaluated as the area-under-the-curve (AUC) or the maximum. In the time domain, a combination of three oscillating decaying exponential functions is used for the fits and the maximum fitted signal used. Due to the high SNR in the presented measurements, fitting could be omitted and signals evaluated directly in the time or frequency domain. All these analysis methods applied to a build-up and decay measurements are shown in Fig. S2. As the line widths in the sample change upon interruption of the MW irradiation (cf. Fig. S2 for frequency domain maximum), the frequency domain maximum is a poor choice as it is not invariant to line width changes. We note that this change of signal is qualitatively different than the observed change in DNP signal for TEMPO in ^{1}H glassy matrices[1] where the signal due to a change in the number of spins detectable through detection pulses (decoupling of electron-nuclear hyperfine coupling by MW irradiation). In the current case, the line width and with it the T_2^* of the FID (Fig. S1) of the detected NMR signal changes while the time domain maximum signal or integrated frequency domain signal are not affected by the change in MW irradiation (Fig. S2). Fitting in the frequency or time domain has the advantage of filtering out noise to some degree which is especially important for low experimental SNRs as encountered at high temperatures or at the outside of the DNP profiles. Fitting in the time domain and extracting the signal from the maximum of the fit (zero FID time) appears as the best choice as it combines filtering of noise if the signal is low, e.g. at high temperatures or at the very outside of the DNP profile, with invariance to a change in line width.

The obtained build-up and decay curves are fitted with either a mono- or bi-exponential function, a square-root exponential (a stretched exponential with $\kappa = 1/2$) or a stretched

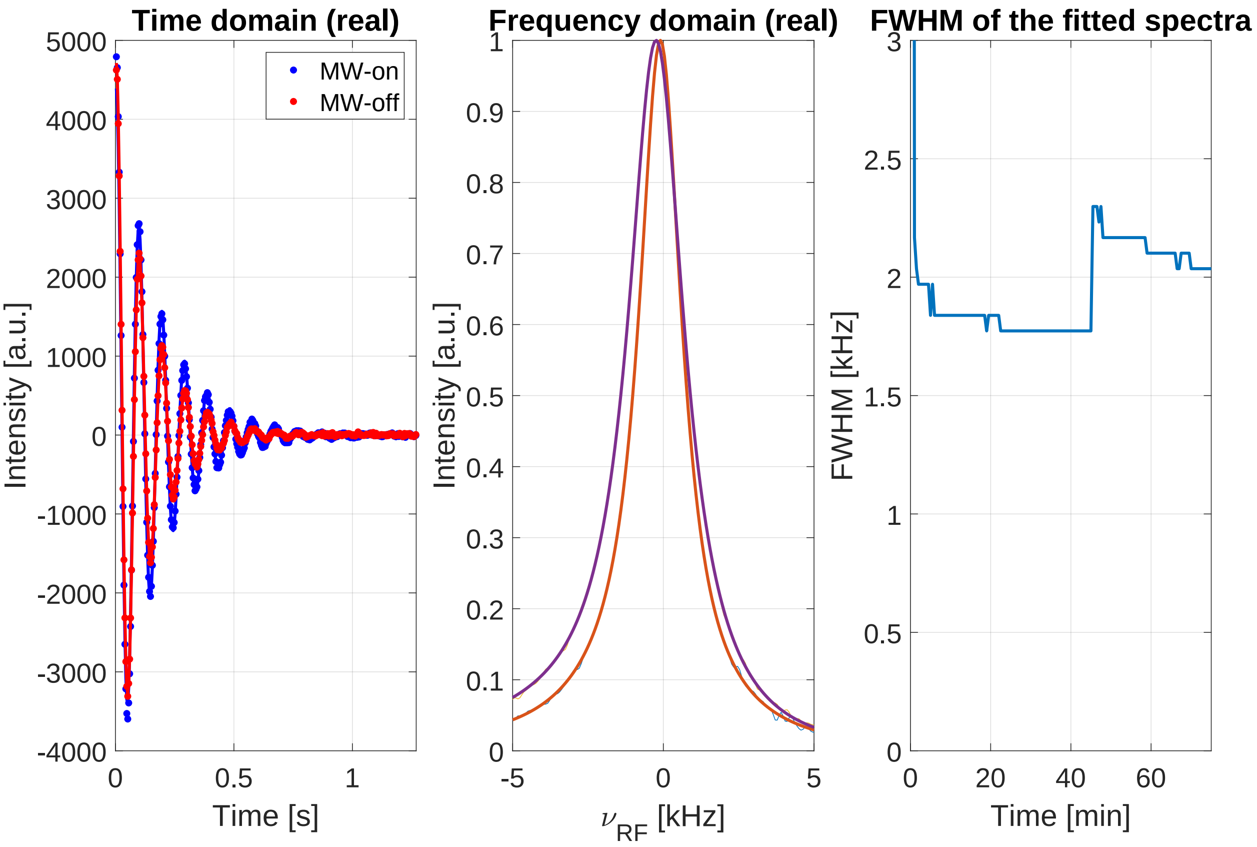

Figure S1: Experimental raw data in the frequency and time domain together with the respective fits. The displayed data belongs to the build-up shown in Fig. S2 at the end of the build-up (MW-on) and right after switching off the MW (MW-off) at 7 T and 3.4 K.

exponential of the form

$$P = P_{0,s} \cdot \left(1 - e^{-(t/\tau_\mathrm{s})^\kappa}\right) \tag{S1}$$

with $P_{0,s}$ being the steady-state polarization of the stretched exponential build-up, τ_s the build-up time constant and κ is the stretch exponent. Eq. (S1) is for the build-up and the decay model is obtained by dropping the $1-$ part. All these build-up and decay fits are shown in Fig. S2.

The stretched and bi-exponential gave the best results throughout this work. We chose a stretched exponential due to its more robust fitted parameters which are given in Figs. 1 and S3. Furthermore, the stretched exponential ansatz has a direct interpretation in terms of a

direct DNP transfer (cf. Sec. S6) while for the bi-exponential ansatz could be interpreted as spin diffusion between different compartments causing different time scales. The latter is hard to imagine with the high and rather homogeneous distribution of defects in the bulk as well as the estimated fast (with respect to the distances between defects) spin diffusion.

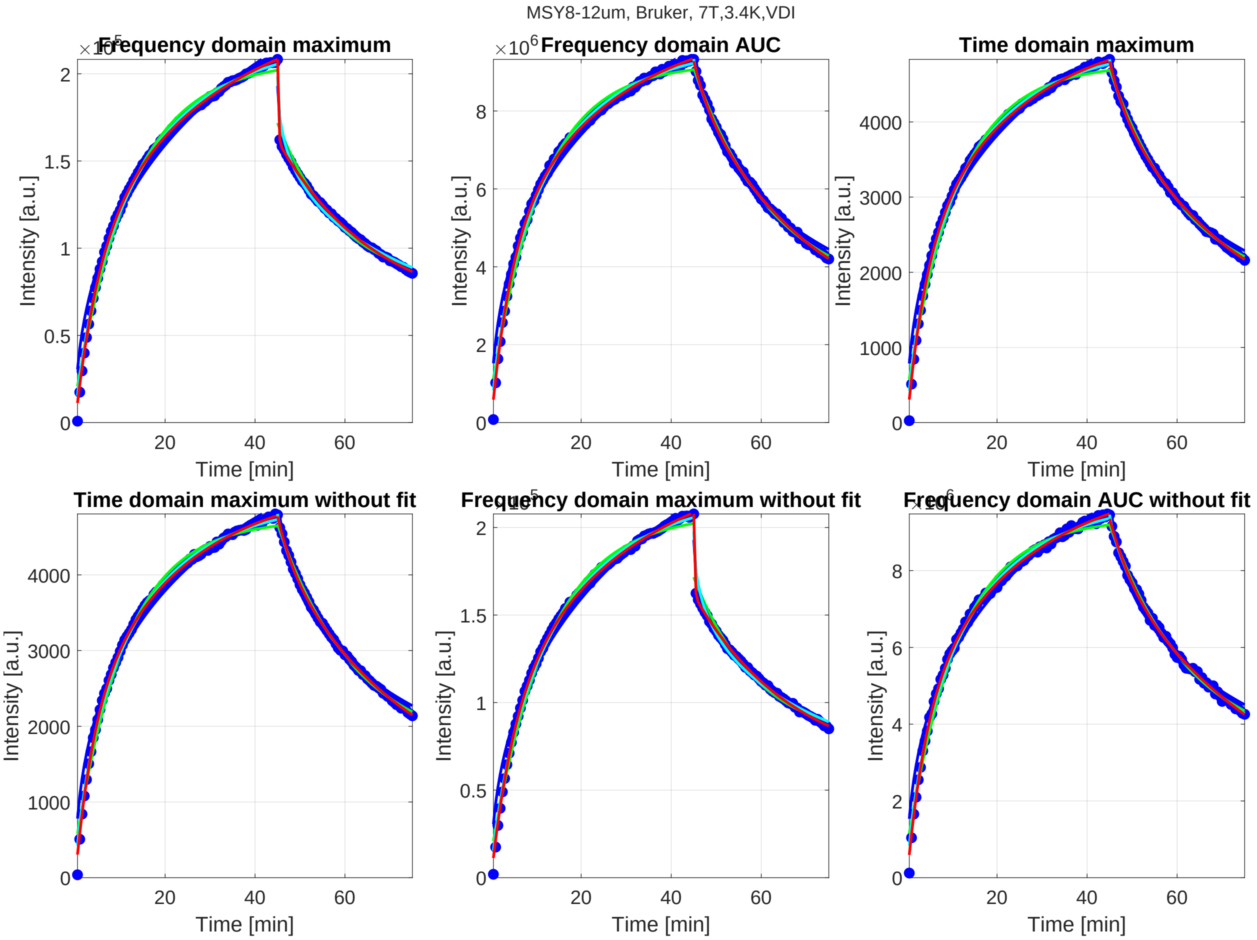

Figure S2: Different analysis methods for the build-up and decay (time or frequency domain, with or without fit, maximum or area under the curve(AUC)) as well as mono- (green), square-root- (a stretched exponential with $\kappa = 1/2$, blue), stretched (cyan) and bi-exponential (red) fits. Data acquired at 7 T and 3.4 K.

For the hyperpolarization decay/ relaxation after the build-up experiment at 7 T and 3.4 K (cf. Fig. 4a) as shown in Figs. 4 and S2, we find a decay time constant $\tau_s^{dec} = 27 \pm 4$ min and a stretch exponent of $\kappa = 0.79 \pm 0.03$. The stretch exponent is in good agreement with the build-up (cf. Figs. S3b and S4c). The stretched exponential decay time constant is nearly twice as long as for the build-up which is attributed to the the vanishing DNP injection with

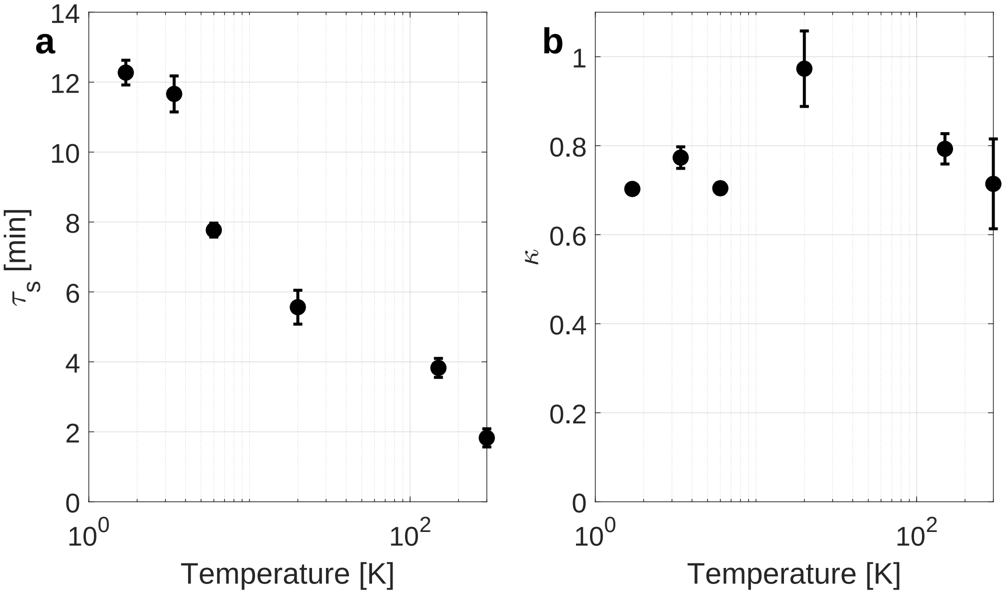

Figure S3: Stretched exponential build-up fit parameters **(a)** τ_s and **(b)** κ (cf. Eq. (S1)) at 7 T. The steady-state polarization for all the temperatures are given in Fig. 1. Uncertainties might be smaller than symbols.

the MW being switched off. For more information on this, the interested reader is referred to Sec. S6.

At 3.4 T and 3.5 K, the stretch exponent for the build-up and decay are 0.59 ± 0.12 and 0.71 ± 0.03. The corresponding stretched exponential time constants for the build-up and decay 6.3 ± 1.5 and 6.9 ± 0.3 min.

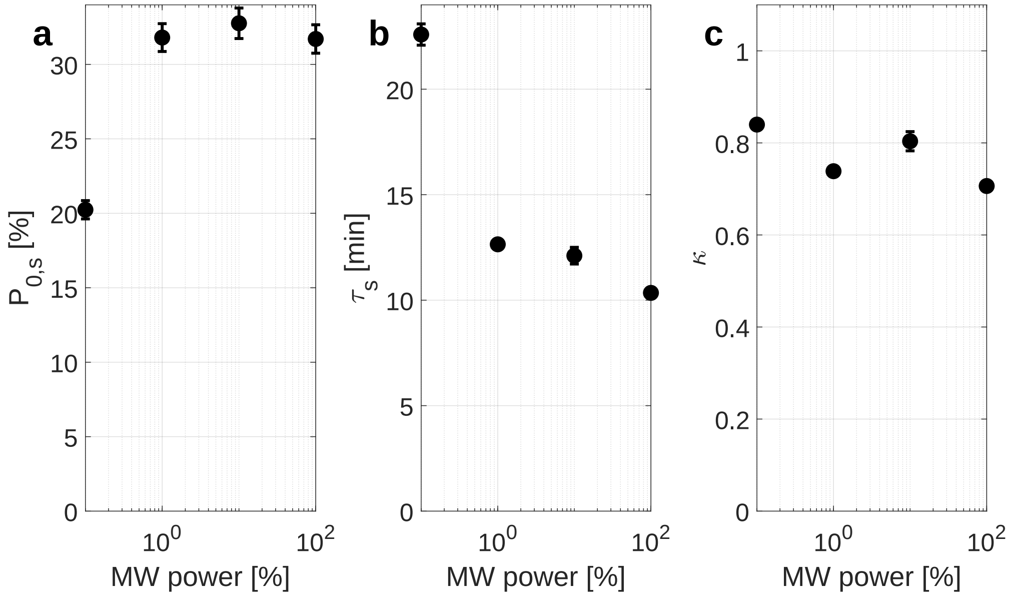

Figure S4: Fit parameters of the data shown in Fig. 4c at 7 T and 3.4 K. The build-ups were fit with a stretched exponential ansatz (cf. Eq. (S1)). The exact MW power for the lowest MW power is unknown but $\ll 1\%$.

S2 DNP profiles and DNP at cryogenic temperatures

Fig. S5 shows the full set of recorded DNP profiles at different temperatures and at 3.4 T and 7 T (cf. Fig. 2).

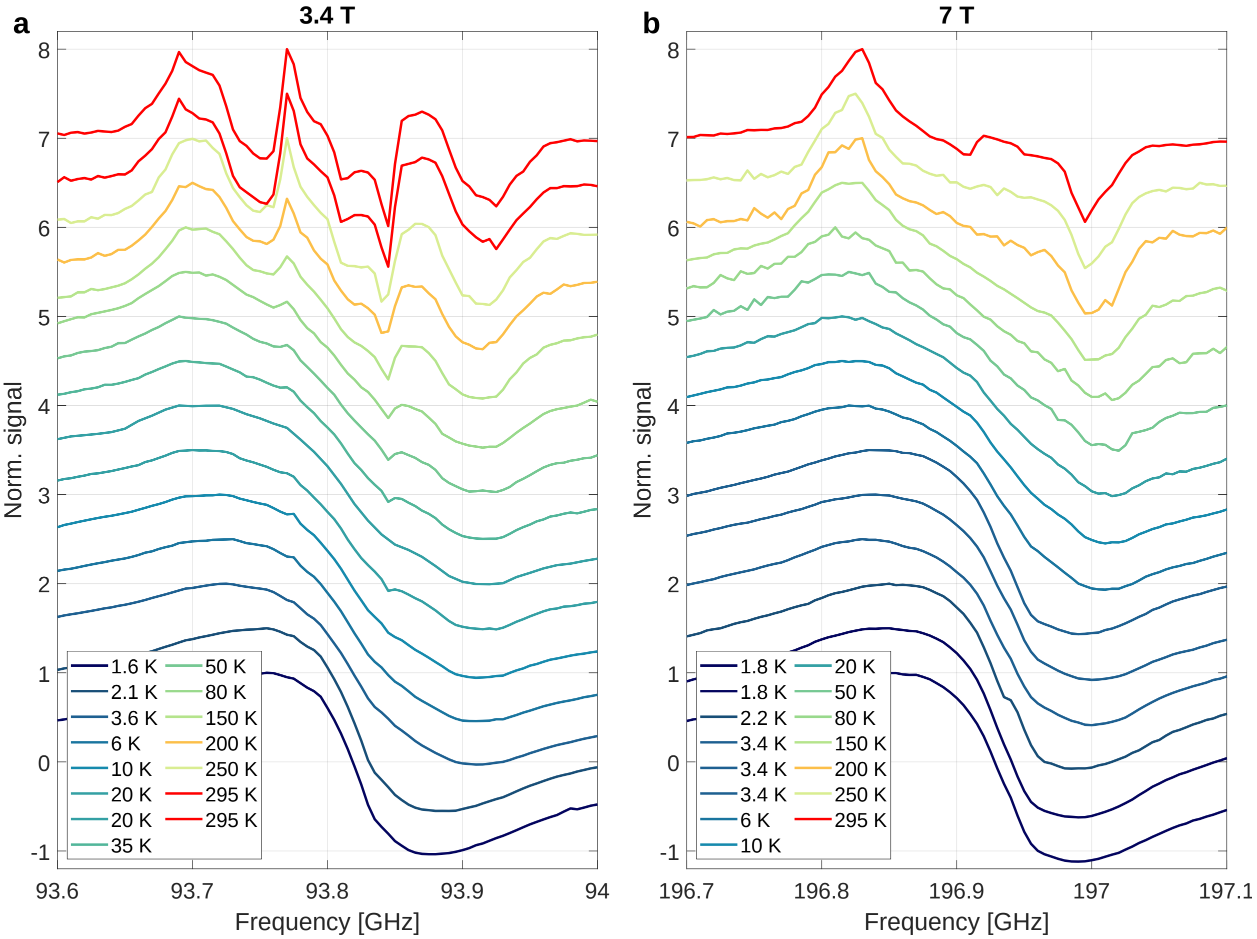

Figure S5: DNP profiles between 295 K and 1.6 K for **(a)** 3.4 T and **(b)**) 7 T. DNP profiles are vertically offset by 0.5 for clarity.

The frequency difference between the DNP maximum and minimum $\Delta\nu_{\mathrm{pp}}$ are shown in Fig. S6 for all DNP profiles from Fig. S5. At 3.4 T (Fig. 2a of the main part), the initial increase of $\Delta\nu_{\mathrm{pp}}$ is explained through the different temperature dependences of the two large inner DNP peaks (around 93.77 GHz and 93.85 GHz) compared to the two large outer DNP peaks (around 93.70 GHz and 93.92 GHz). Owing to the broad DNP profiles, a slight variation between separate measurements exists, e.g. at 3.4 K at 7 T (cf. Fig. S6a). At 7 T and liquid-helium temperatures, the two DNP peaks appear approximately $2\omega_{\mathrm{n}}(7\ \mathrm{T}) \approx$

$2 \cdot 75$ MHz $= 150\,$MHZ apart (cf. Fig. S6a), which would indicate a solid effect (SE) from a broad electron line. However, at 3.4 T the frequency difference between the DNP maximum and minimum is in a similar range, which is much larger than the $2\omega_n(3.4\ \mathrm{T}) \approx 2 \cdot 36\,$MHz.

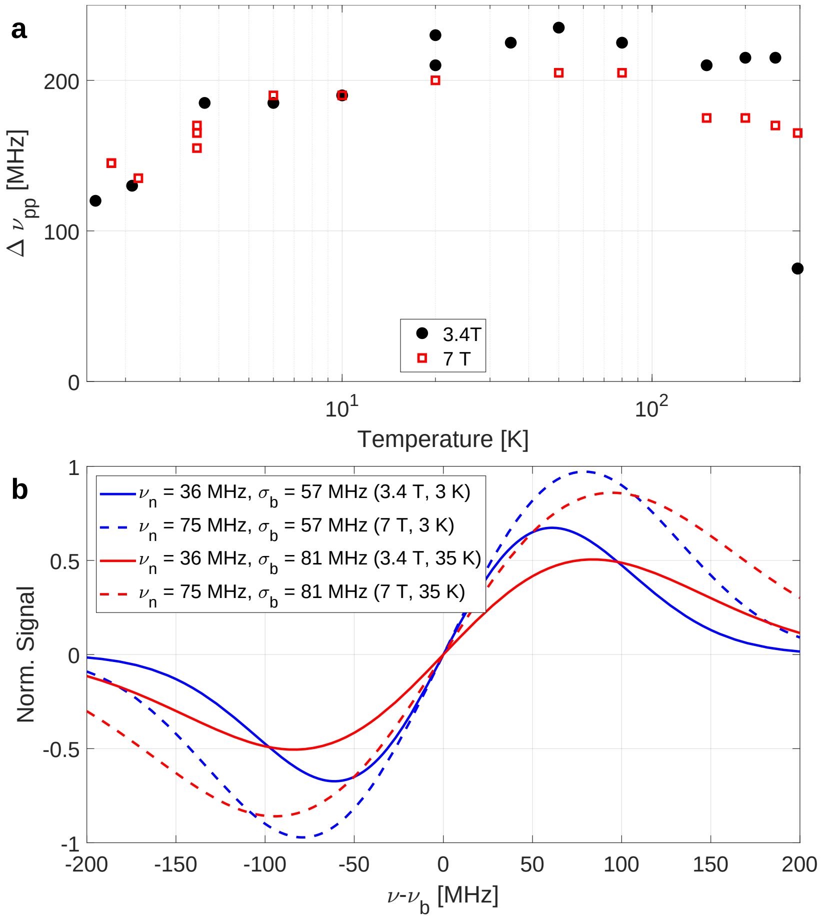

Figure S6: **(a)** Frequency difference between the maximum and minimum of the DNP profiles ($\Delta\nu_{\mathrm{pp}}$) depending on the temperature for 3.4 T and 7 T. The corresponding DNP profiles are show in Fig. S5, Supporting Information. Experimental uncertainties can only be estimated from conditions for which multiple measurements have been performed, e.g. at 3.4 K and 7 T, and, hence, are not shown. **(b)** Evaluation of Eq. S2 for different broad electron line widths σ_{b} (as observed at different temperatures in Fig. 3f) and different resonance frequencies. The electron line broadening from 7 T is used for both fields. For an assumed solid effect from the broad electron line at low temperatures, the frequency difference between the DNP maximum and minimum decreases for lower temperatures and is similar at 3.4 T and 7 T.

To better understand if the broad DNP lines at cryogenic temperatures might arise from

a solid effect from the broad electron line, we can evaluate

$$S_{\text{DNP}} = \frac{e^{-\frac{(\nu+\nu_{\text{n}})^2}{2\sigma_{\text{b}}^2}} - e^{-\frac{(\nu-\nu_{\text{n}})^2}{2\sigma_{\text{b}}^2}}}{e^{-\frac{1}{2\sigma_{\text{b}}^2}}} \tag{S2}$$

for different line broadenings σ_{b} at different temperatures (cf. Fig. 3f) and nuclear Larmor frequencies ω_n. This is shown in Fig. S6b. This approach qualitatively reproduces two key features from the experiments. First, the frequency difference $\Delta\nu_{\text{pp}}$ is similar for 3.4 T and 7 T owing to the electron line width being broader or similar to ω_{n}. Second, $\Delta\nu_{\text{pp}}$ increases between 3.3 K and 35 K due to an increase of σ_{b}. Therefore, the broad DNP line at cryogenic temperatures in diamond might arise from the broad electron line, which could be due to N2 or N3 centers. However, the insufficient understanding of the electronic spin system in diamond hinders any understanding of the DNP processes and completely different explanations might be feasible.

In Eq. S2 and with it Fig. S6b, the maximum and minimum signal is normalized to 1 and, hence, describes the fraction of the broad electron line maximum that can contribute to the net DNP enhancement at a given frequency. For example, for 3.4 T and 3.3 K, at most around 60% of the broad electron line maximum could contribute to the DNP. This improves to around 97% for 3.3 K and 7 T, partially offsetting the B_0^{-2} scaling of the solid effect.

Fig. S6 only relies on 7 T LOD EPR measurements and it is not clear if the line broadening at the different temperatures translates one-to-one to 3.4 T. Comparing Fig. S6 with the experimental DNP profiles (cf. Figs. 2 and Fig. S5, Supporting Information), it is evident that the simple idea behind Eq. S2 cannot describe the gently decreasing DNP signal on the very outside of the DNP profiles. Thus, a better understanding of the defects in diamond is necessary to understand the DNP at the different temperatures.

S3 Longitudinal-detected (LOD) electron paramagnetic resonance (EPR)

S3.1 LOD profiles at 7 T of 10 µm diamonds

The first type of LOD experiments presented in this study is the MW frequency dependence of the LOD signal (LOD profile or spectrum). Fig. S7 shows the measured LOD profiles between 3.4 K and 295 K fitted with Eq. (1) of the main text consisting of a P1 centers and a broad component (for more details, see main text). The fit parameters are summarized in Fig. 3 of the main part.

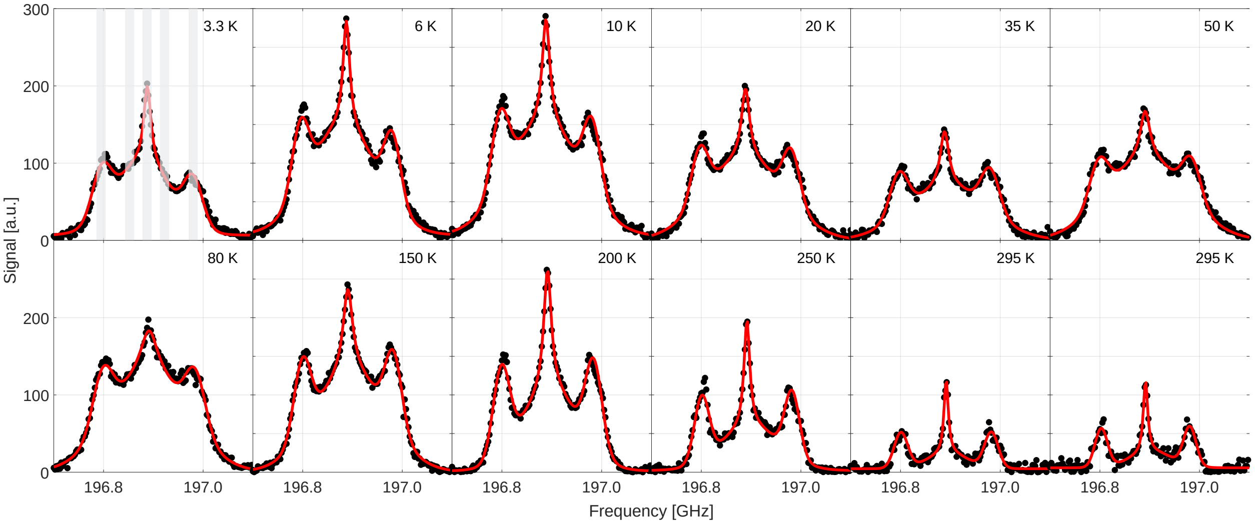

Figure S7: LOD profiles of the 10±2 µm diamonds at different temperatures. The profiles are fitted with a combination of P1 centers (Lorentzian central $m_I = 0$ line shape and Gaussian $m_I = \pm 1$ line shape) and a broad Gaussian line (cf. Eq. (1) of the main text). The fit parameters for these are summarized in Fig. 3 of the main text. In each of the grey shaded frequency intervals, measured τ_{LOD} are averaged and summarized in Fig. S10 (cf. text in Supporting Information for more details).

In addition, the measured LOD EPR data was fitted with an approach motivated by isolated and clustered P1 centers as discussed in[2–4] leading to a narrow and broad P1 spectrum as discussed in the main text, particularly Eq. (2). The fitted spectra in the range of 3.3 K to 295 K are shown in Fig. S8 with the fit parameters summarized in Fig. S9.

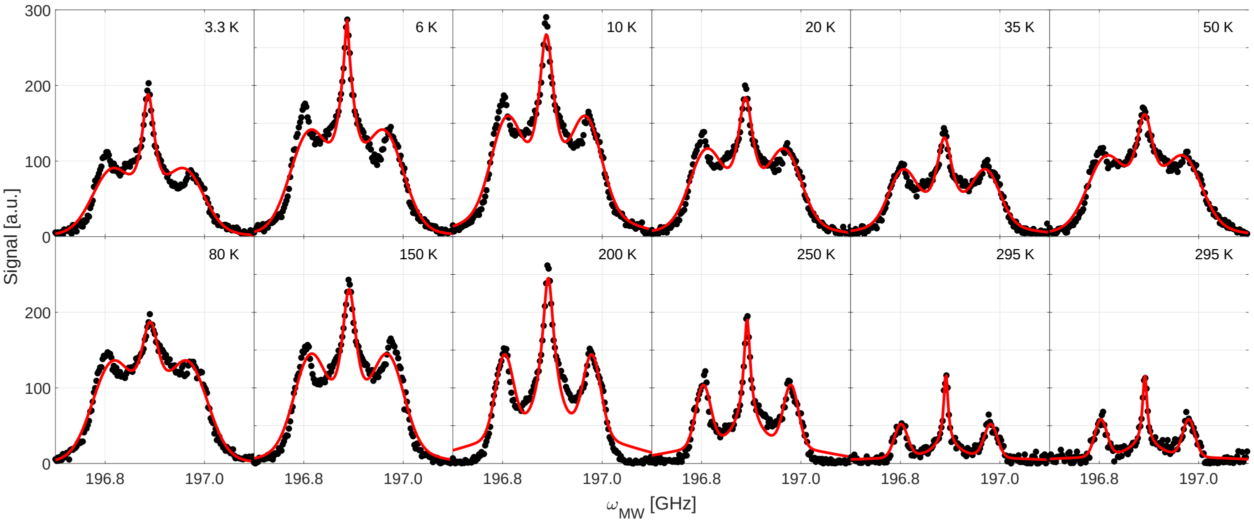

Figure S8: LOD profiles of the $10 \pm 2\,\mu$m diamonds at different temperatures. The profiles are fitted with a combination of two different types of P1 centers (Lorentzian central $m_I = 0$ line shape and Gaussian $m_I = \pm 1$ line shape) as described by Eq. (2): narrow isolated and cluster-broadened broad P1 centers as suggested in Ref.[2–4] . The fit parameters for these are summarized in Fig. S9.

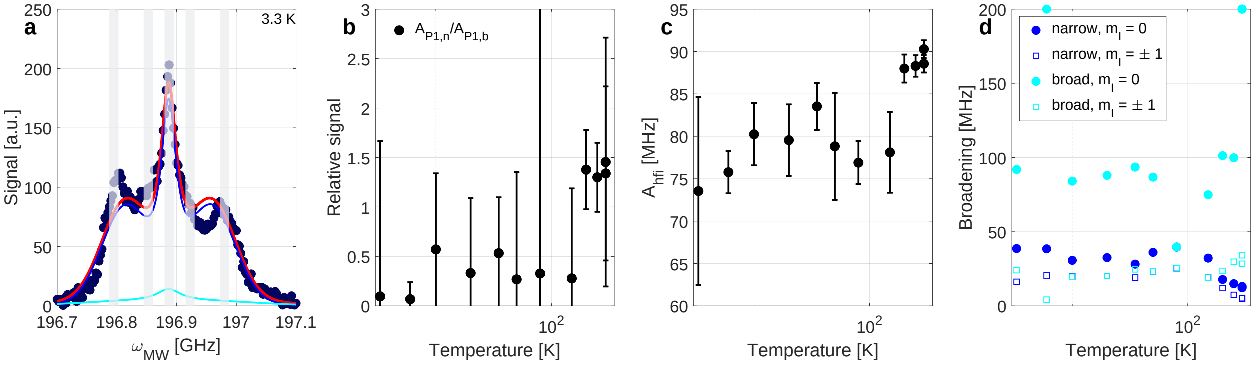

Figure S9: Summary of the fit parameters from Fig. S8 of the $10\pm2\,\mu$m diamonds at different temperatures. The profiles are fitted with a combination of two different types of P1 centers (Lorentzian central $m_I = 0$ line shape and Gaussian $m_I = \pm 1$ line shape) as described by Eq. (2): narrow isolated and cluster-broadened broad P1 centers as suggested in Ref.[2–4]

S3.2 LOD decay times at 7 T of 10 µm diamonds

LOD EPR measures the induced voltage after the polarization of the electrons is changed either upon switching the MW on or off (this has similarities to a $T^*_{2,\mathrm{n}}$ experiment in NMR). Our LOD EPR experiments consist of two parts: (i) The MW is switched on, saturating the electron line at the given frequency and through electron spectral diffusion (eSD) to neighbouring frequencies. (ii) The MW is switch off and the electron system relaxes back to thermal equilibrium. These two parts are denoted as 'MW on' and 'MW off' in Fig. S10. The signal decays in $< 1\,\mathrm{ms}$ (cf. Fig. S10) - consistent with other materials used in DNP.[5] The MW on and off parts are each fitted with a mono-exponential decay with a decay constant τ_{LOD}. Owing to the rather low SNR, the values of τ_{LOD} for a single data point fluctuate. Averaging over a narrow frequency interval gives more reliable values.

We note that LOD EPR does not measure $T_{1,\mathrm{e}}$ but rather $\tau_{\mathrm{LOD}} = \left(\frac{1}{T_{1,\mathrm{e}}} + \frac{1}{\tau_{\mathrm{ff}}}\right)^{-1}$ with τ_{ff} being an electronic flip-flop time describing the electron couplings and with it electron spectral diffusion.

For five different frequency intervals which are shaded in gray in Fig. S7 for 3.3 K, the obtained averaged LOD time constants τ_{LOD} are shown in Fig. S10. The trend is for all five frequencies similar: At low temperatures τ_{LOD} is around 500-600 µs with the 'MW on' time slightly shorter. τ_{LOD} is rather stable up to temperatures of a few tens of Kelvin before shortening to around 200 µs at room temperature. The measured LOD time constants appear frequency independent (cf. Fig. S10), indicating electron-electron cross relaxation between the P1 and broad electron lines. Decreasing the temperature from room temperature to liquid-helium temperatures monotonically increases τ_{LOD} from around 200-300 µs to around 500-600 µs. Assuming that $T_{1,\mathrm{e}} \gg \tau_{\mathrm{LOD}}$ at liquid-helium temperatures, the measured time constant would describe the electronic flip-flop time τ_{ff}. For $\tau_{\mathrm{LOD}} \approx 300\,\mu\mathrm{s}$ at room temperature and $\tau_{\mathrm{ff}} \approx 600\,\mu\mathrm{s}$ from the low temperature LOD EPR data (cf. Fig. S10), $T_{1,\mathrm{e}} \approx 600\,\mu\mathrm{s}$ at room temperature which is shorter than the typically reported $\approx 2\,\mathrm{ms}$[6,7] for P1 centers at room temperature.

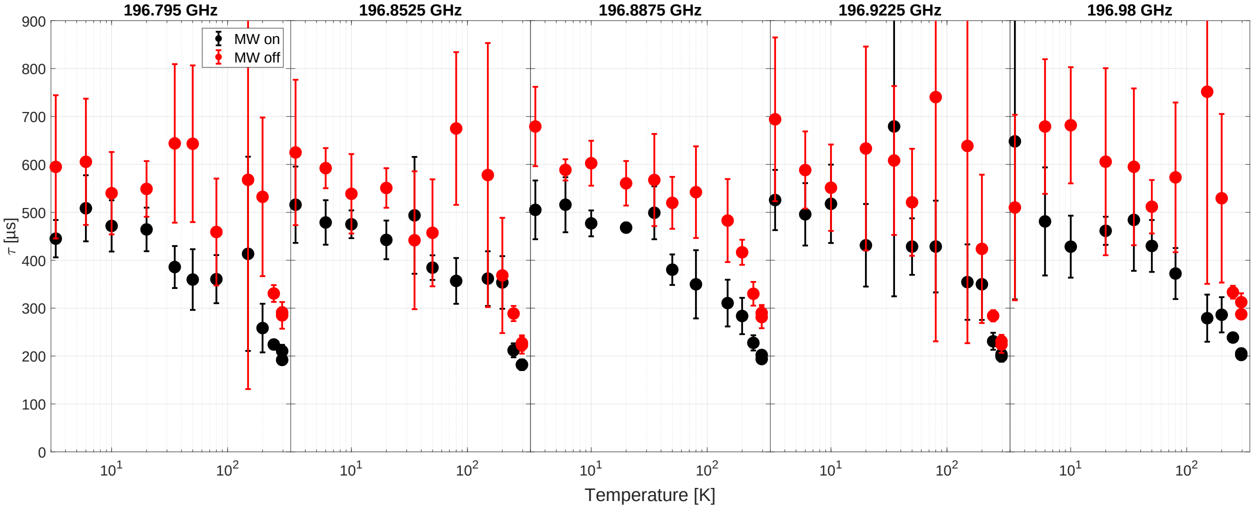

Figure S10: Electronic relaxation times τ_{LOD} in LOD EPR at 7 T for different temperatures and frequency intervals sketched by the grey shading in Fig. 3a. Each data point is an average over five frequencies. Uncertainties are derived from the standard deviation of the five frequencies.

S3.3 Power dependence at 7 T of 10 µm diamonds

Another group of LOD EPR measurements concerns the power dependence of the signal. LOD EPR power measurements for temperatures between 3.3 K and 295 K are shown in Fig. S11. For the power curves, the measured signal of 'MW off' part was numerically integrated (summed over the data points). The resulting power curves are fitted with the time-independent part of the Torrey model of damped Rabi oscillations which is equivalent to the z-component of the time-independent Bloch equations.[8] Specifically, the model is given by

$$1 - \frac{P_{e,\infty}}{P_{0,e}} = 1 - \frac{1}{\gamma_e^2 B_{1,\mathrm{MW}}^2 T_{2,\mathrm{e}} T_{1,\mathrm{e}} + 1} \qquad \text{(S3)}$$

with the electronic relaxation times $T_{1,\mathrm{e}}$ (spin-lattice) and $T_{2,\mathrm{e}}$ (spin-spin). $B_{1,\mathrm{MW}}$ is the magnetic field generated by the MW perpendicular to the main magnetic field B_0.

If the conversion from MW power to $B_{1,\mathrm{MW}}$ for a given experimental set-up is known, LOD EPR might be used to measure $T_{1,\mathrm{e}}$ and $T_{2,\mathrm{e}}$ as relevant for DNP. In the current case, a simplified expression was used (cf. captions of Figs. S11 and S12) and the best fit parameters

are shown in Fig. S12. We note that the Torrey model is able to explain the nearly linear LOD EPR signal-power data found in Ref.[5]

The amplitude of the signal does not follow the thermal electron polarization, which increases by around two orders of magnitude between the highest and lowest temperature. In contrast, the amplitude of the LOD signal only increases around fourfold. At high temperatures, the applied MW power is almost enough to fully saturate the electron line (the measured signal is close to the fitted maximum signal) while at low temperatures eventually only around half the electron line is saturated (at 10 K the saturation seems the most difficult). The b parameter describing the electron relaxation is nearly independent of the temperature with values around 0.02-0.03 for all temperatures except 80 K.

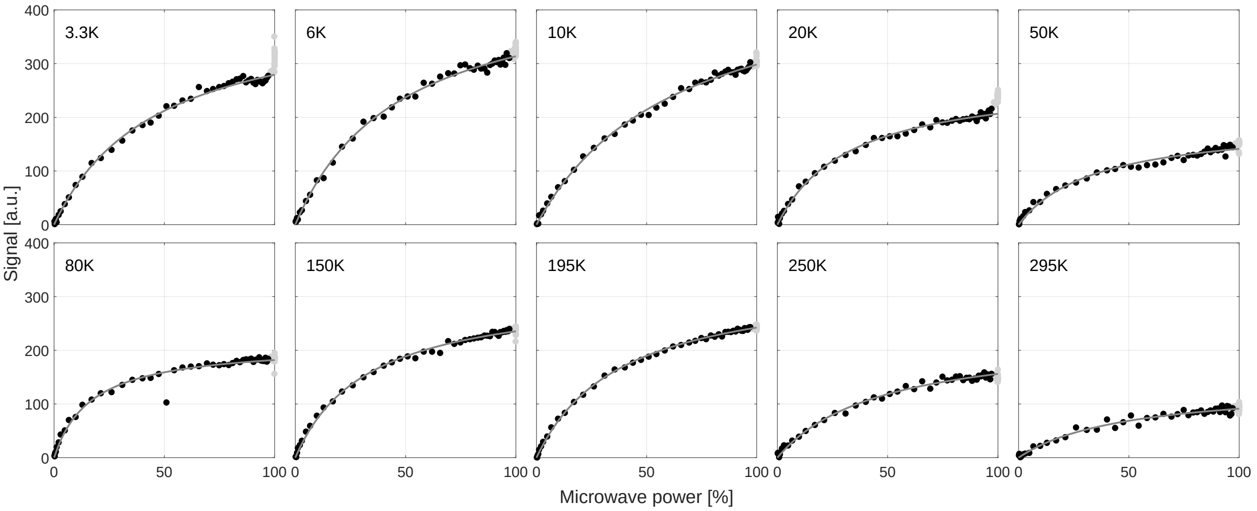

Figure S11: Power dependence of the LOD EPR signal at 7 T, 196.905 GHz for different temperatures. The data is fitted with $a\left(1-\frac{1}{bx+1}\right)$ (cf. Eq. (S3)) with $bx=\gamma_e^2 B_{1,\mathrm{MW}}^2 T_{2,\mathrm{e}} T_{1,\mathrm{e}}$ being the saturation factor (x is the MW power which defines $B_{1,\mathrm{MW}}$).[8] The best fit parameters are shown in Fig. S12.

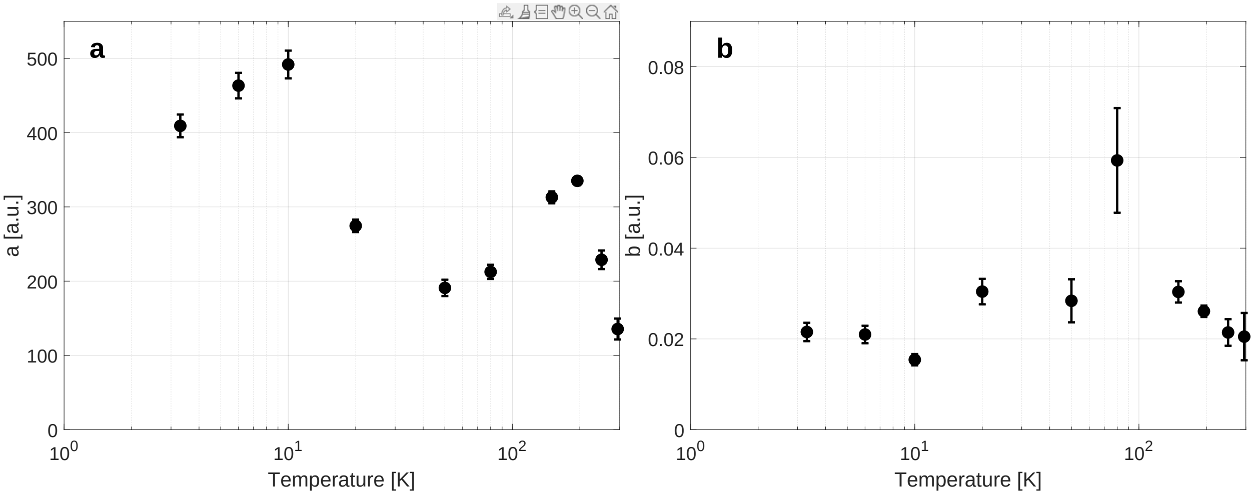

Figure S12: Best fit parameters of the power dependence of the LOD EPR signal at 7 T, 196.905 GHz for different temperatures as shown in Fig. S11. The data is fitted with $a\left(1-\frac{1}{bx+1}\right)$, derived from the simplified Torrey model (cf. Eq. (S3)).[8] The data points for the highest MW powers are omitted (shown in light gray) for the fitting process as problems with setting the MW occurred at the beginning of each measurement (the highest powers were measured first). Uncertainties might be smaller than the symbols.

S3.4 LOD profiles of nanodiamonds

Fig. S13 shows the fitted LOD profile of a nanodiamond sample (< 10 nm) at 7 T and 20 K. We immediately note the absence of the three P1 peaks as expected for small nanodiamonds with with a large surface-to-volume ratio. To fit the measured spectrum, we use

$$S_{\mathrm{EPR}} = \frac{S_{\mathrm{b}}}{\sqrt{2\pi}\sigma_{\mathrm{b}}} e^{\frac{(\nu-\nu_{\mathrm{b}})^2}{2\sigma_{\mathrm{b}}^2}} + \frac{S_{\mathrm{n}}}{\sqrt{2\pi}\sigma_{\mathrm{n}}} e^{\frac{(\nu-\nu_{\mathrm{n}})^2}{2\sigma_{\mathrm{n}}^2}} + S_{\mathrm{offset}} \tag{S4}$$

with a broad and narrow spin-1/2 Gaussian electron line. Details about the fit parameters are found in Tab. S1. The broad and narrow spin-1/2 components are discussed in more detail below.

Table S1: Fit parameters of the LOD spectrum shown in Fig. S13 and fitted with Eq. (S4).

	broad	narrow
Weight [%]	63(12)	37(12)
Frequency [GHz]	196.942(6)	196.970(3)
Line Width [MHz]	72(5)	40(4)

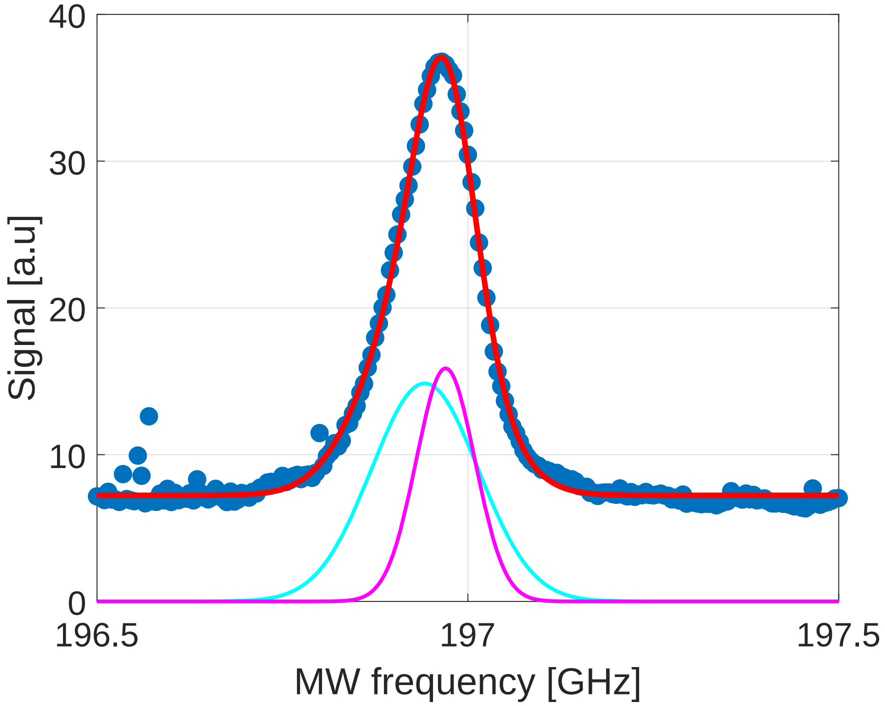

Figure S13: LOD EPR spectrum of nanodiamonds ($< 10\,\text{nm}$) sample at $7\,\text{T}$ and $20\,\text{K}$. The spectrum is fitted with Eq. (S4). The fit parameters can be found in Tab. S1.

Fig. S14 shows the fitted LOD profile of diamonds smaller than $250\,\text{nm}$ at $3.4\,\text{T}$ and $20\,\text{K}$. In contrast to the larger $10\,\mu\text{m}$ diamonds at $7\,\text{T}$, an additional rather narrow electron line emerges at a frequency roughly $25\,\text{MHz}$ higher than the $m_I = 0$ line of the P1 center. To fit the measured spectrum, we added another term to Eq. (1) to describe the rather narrow line (denoted with "n" subscript)

$$S_{\text{EPR}} = \frac{S_{\text{P1}}}{\sqrt{2\pi}\sigma_{\pm 1}}\left[e^{-\frac{(\nu-(\nu_{\text{P1}}-A_{\text{P1}}))^2}{2\sigma_{\pm 1}^2}} + e^{-\frac{(\nu-(\nu_{\text{P1}}+A_{\text{P1}}))^2}{2\sigma_{\pm 1}^2}}\right]$$
$$+ \frac{S_{\text{P1}}}{\pi}\frac{\sigma_0}{(\nu-\nu_{\text{P1}})^2+\sigma_0^2} + \frac{S_{\text{b}}}{\sqrt{2\pi}\sigma_{\text{b}}}e^{\frac{(\nu-\nu_{\text{b}})^2}{2\sigma_{\text{b}}^2}}$$
$$+ \frac{S_{\text{n}}}{\sqrt{2\pi}\sigma_{\text{n}}}e^{\frac{(\nu-\nu_{\text{n}})^2}{2\sigma_{\text{n}}^2}} + S_{\text{offset}} \tag{S5}$$

Details about the fit parameters are found in Tab. S2. Similar to $7\,\text{T}$, the broad component is the largest contributor to the signal at low temperatures, is centered within a few MHz of the $m_I = 0$ P1 line and has a similar line width at $3.4\,\text{T}$. The results for the broad and

narrow spin-1/2 component in terms of center frequencies and line widths are similar to < 10 nm diamonds at 7 T (cf. Fig. S13 and Tab. S2). However, the < 10 nm diamonds with a much higher surface-to-volume ratio have a more similar intensity ratio between the broad and narrow components. Furthermore, the narrow component is not observed for the larger 10 µm diamonds. This suggests that the narrow spin-1/2 component arises from surface defects while the broad spin-1/2 component arises from a different type of bulk defect than P1 centers.

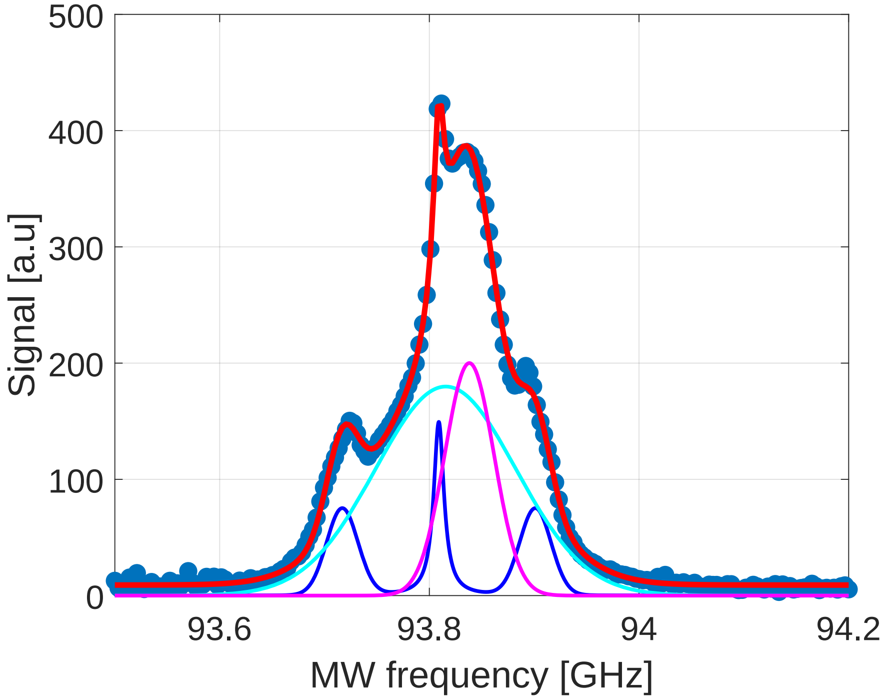

Figure S14: LOD EPR spectrum of diamonds smaller than 250 nm studied at 3.4 T and around 20 K. The spectrum is fitted with Eq. (S5). The fit parameters can be found in Tab. S2.

Table S2: Fit parameters of the LOD spectrum shown in Fig. S14 and fitted with Eq. (S5).

A_{P1} [MHz]	P1<br>92.1(7)	broad	narrow
Weight [%]	6.4(7)	68(3)	25(3)
Frequency [GHz]	93.809(3)	93.816(2)	93.838(1)
Line Width [MHz]	15.0(9) ($m_I = \pm 1$), 6.0(5) ($m_I = \pm 0$)	67(2)	24(1)

S4 X-band EPR

In addition to the LOD EPR data, we performed X-band EPR (335 mT) to estimate the number of electron spins and to enable a comparison to previous works on diamond particles.[9–11] For this, we compared the $10 \pm 2\,\mu$m sample used throughout this work with $2 \pm 0.5\,\mu$m, < 250 nm and < 10 nm diamond particles (cf. Sec. S3). The measured X-band EPR spectra are shown in Fig. S15. The spectra are fitted with EasySpin[12] with a combination of broad and narrow spin-1/2 defects as well as P1 centers similar to Ref.[9–11] The total number of electron spins with respect to TEMPO and porous silicon samples are shown in Fig. S16. The spin concentration decreases with increasing particle size. With increasing particle sizes, the fraction of P1 centers increases. For the $10\,\mu$m diamonds, an estimated 58% of 54 ppm of electron spins are P1 centers. We notice that the Gaussian broadening in the $10 \pm 2\,\mu$m-sized diamonds is noticeably larger than in the other samples (cf. Fig. S16d) and appears in agreement with the broadening of the broad component in LOD EPR at 295 K (cf. Fig. 3f).

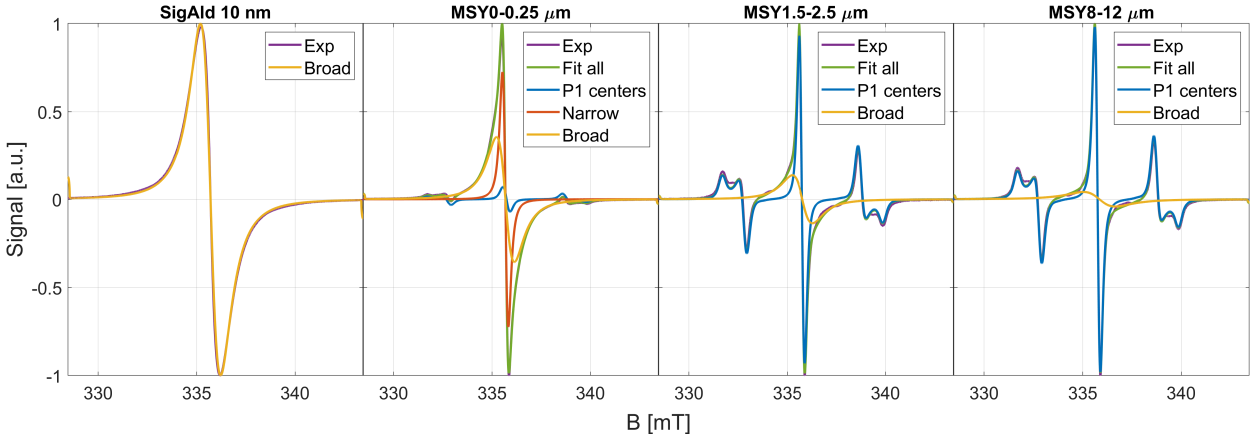

Figure S15: Measured and fitted X-band EPR spectra of four different diamond samples at room temperature. The spectra are fitted with EasySpin and a combination of broad and narrow spin-1/2 defects as well as P1 centers. The assumed spin system for the fit models is described by the legends.

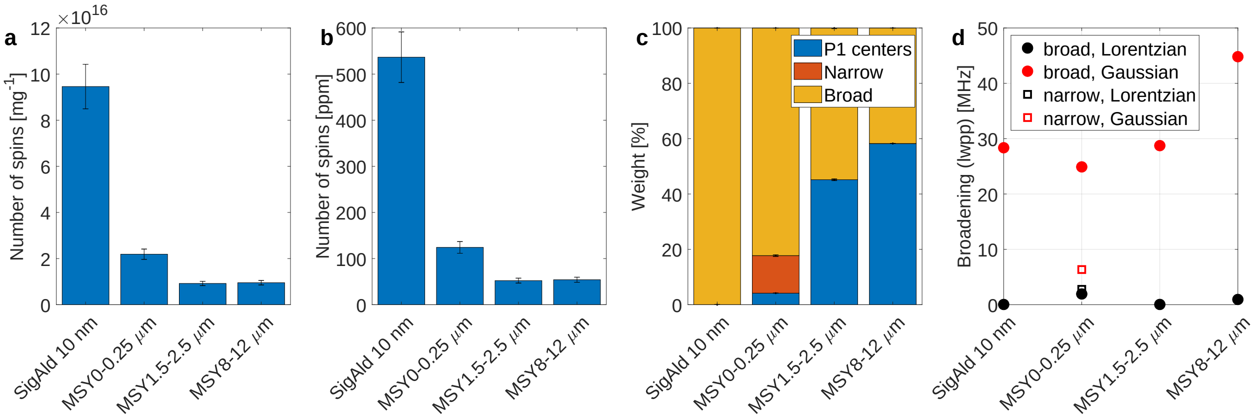

Figure S16: **(a)** Number of electron spins per mg or **(b)** converted to ppm in the different diamond samples. **(c)** Relative weights of the different spin systems in percent of the total number of spins as displayed in a,b. The assumed spin system for the fit models is described by the legends in Fig. S15. **(d)** Lorentzian and Gaussian line broadening of the narrow and broad components.

S5 Discussion of possible defects

The g-factor agreement of the broad and narrow line observed with high-field EPR[2,3] supports the proposed clustering of P1 centers. These clusters provide a pool of fast-relaxing electron spins required for truncated cross effect (tCE) DNP, which seems to dominate DNP at high fields and room temperature.[2,3] As discussed above, this explanation does not appear compatible with the asymmetric low-temperature LOD EPR measurements reported herein. Moreover, as will be discussed below, the electronic relaxation times of samples containing only P1 centers at cryogenic temperatures appear incompatible with the observed nuclear hyperpolarization build-up times. However, we cannot fully rule out the possibility of P1 clusters. Below, we discuss alternatives to the cluster ansatz, which seem to be in better agreement with our data.

X-band[9,10] and Q-band[11] EPR spectra of diamond nano- and microparticles specifically manufactured for hyperpolarized MRI applications were fitted with an ansatz of P1 centers, broad- and narrow spin-1/2 electron lines. The broad spin-1/2 component is ascribed to surface defects while the narrow is ascribed to bulk defects. At Q-band, the fraction of P1 and broad spin-1/2 defects are size-dependent with larger particles containing more (bulk) P1 defects and fewer surface defects owing to the higher volume-to-surface ratio.[11] However, the narrow spin-1/2 defects' fraction shows a stronger size dependence than the broad spin-1/2 electron line (except for natural diamonds) leaving some ambiguity if (only) the broad component is really arising from surface defects. An additional open question are the rather short cryogenic $T_{1,\mathrm{e}}$ of at most tens of milliseconds - orders of magnitude shorter than the tens of seconds reported for P1 centers at X-band in Ref.[13] (see above). This raises the question what exactly the broad- and narrow spin-1/2 electron lines are and how these interact with the P1 centers.

In Sec. S3, Supporting Information, LOD EPR at 3.4 T and 7 T of different diamond sizes is analyzed, which we will briefly summarize here. For nanodiamonds smaller than 250 nm, the LOD EPR spectrum resembles that of the microdiamonds discussed throughout this

work (cf. Figs. 3 and S7, Supporting Information) apart from an additional narrow spin-1/2 component at a roughly 20 MHz higher center frequency compared to the P1 $m_I = 0$ line. For nanodiamonds smaller than 10 nm, the P1 lines are no longer visible and the narrow signal has a higher relative signal contribution than for the larger nanodiamonds. We note that for both samples and fields the broad spin-1/2 electron line is centered within a few MHz of the $m_I = 0$ line of the P1 center and has a line width around 70 MHz at 20 K. Combining this with the absence of any narrow spin-1/2 electron line for the microdiamonds suggests that the narrow spin-1/2 electron line belongs to surface dangling bonds while the broad spin-1/2 electron line describes some type of bulk defect.

The X-band EPR results presented in Sec. S4, Supporting Information, are somewhat at odds with the LOD EPR analysis: For nanodiamonds smaller than 10 nm, a single defect is found at room temperature with a roughly 30 MHz Gaussian broadening. For larger diamonds, P1 centers are evident and an additional line with less than 10 MHz broadening is fitted for the nanodiamonds smaller than 250 nm. Except from the 10 µm diamonds, the spin-1/2 component has a broadening of around 30 MHz. For the 10 µm diamonds, the broadening is around 45 MHz at room temperature and with this consistent with the LOD EPR results shown in Fig. 3f. Hence, it appears possible that the broad line in the 10 µm diamonds arises from another form of bulk defects. The presence of a different bulk defect species could explain the rather short electronic relaxation times, (LOD) EPR profiles and eventually the DNP profiles. Furthermore, the rather large particle size of 10 ± 2 µm, makes it rather unlikely that around 42% of the defects in the sample (cf. Sec. S4, Supporting Information) arise from surface defects (low surface-to-volume ratio). Future studies might clarify the nature of the different defects in the diamond samples and how these influence DNP.

In the following, we will discuss a selected number of nitrogen-based bulk defects in diamond that could be consistent with the results presented in this work. Specifically, we will discuss three possible nitrogen defects previously studied together with the P1 center

which posses similar g-factors as the P1 or at least overlapping EPR lines at X- ($\approx 0.3\,$T) or W-band ($\approx 3.4\,$T) as possible candidates to explain the broad spin-1/2 component. We note that a much larger range of defects in diamond is known and Ref.[14] might provide a first overview to the interested reader although information on some of the defects got refined in subsequent years. Below, we follow the nomenclature from Refs.[7,13,15] in naming the different defects as some ambiguity in naming between different publications exists.

- The first nitrogen defect we discuss in some more detail besides the P1 center is the so-called P2 center which consists of three substituional nitrogen atoms and a vacancy (N_3V). The P2 center has a broad EPR line owing to its large number of energy levels and overlaps only with the P1's $m_I = 0$ EPR line at X- and W-band.[6,15] The P2 center has a long and field-independent $T_{1,\mathrm{e}}^{\mathrm{P2}} \approx 2.2\,$ms at room temperature[6] which is similar to the $T_{1,\mathrm{e}}^{\mathrm{P1}}$ at room temperature.[13] If P1 and P2 centers are present in the same sample, they tend to cross-relax such that both have a similar temperature dependence with $T_{1,\mathrm{e}}$ on the order of a few milliseconds at room temperature and seconds to hundreds of seconds below 10 K.[13] At low temperatures, a higher P1 concentration, i.e. 0.2 vs. 95 ppm, leads to a shorter $T_{1,\mathrm{e}}$ on the order of several seconds.[13]

- The N2 center (called W7 center in Ref.[14]) is considered to consist of a N-C-N or N-C-C-N complex.[7,14,16] Its properties are temperature-dependent due to a dynamic Jahn-Teller effect with a rather low activation energy[16] leading to an estimated room temperature spin-lattice relaxation time of a few nanoseconds.[7] The N2 center shows a broad central line and six weaker hyperfine lines on either side of the central line.[14,16]

- The N3 center consists of substitutional N and O atoms[17]) with a slight g-factor anisotropy at room temperature[17] which could explain the rather broad electron line observed in our LOD experiments. Strong cross-relaxation with P1 centers was previously observed[13] causing an unexplained shortening of $T_{1,\mathrm{e}}^{\mathrm{P1}}$ with a pronounced asymmetry between different P1 hyperfine contributions reaching $T_{1,\mathrm{e}}^{\mathrm{P1},\ m_I=0}/T_{1,\mathrm{e}}^{\mathrm{P1},\ m_I=-1} \approx 100$

below 10 K.[13]

As discussed in the main part, P2 centers appear rather inefficient in shortening the electronic relaxation time of P1 centers. Therefore, N2 and N3 centers appear to be the more likely candidates for the broad electron line although further research is necessary.

S6 Uncoupled compartments model of DNP

We start with a recap of the previously introduced single homogeneous compartment model:[18] The hyperpolarization build-up can be described through a first-order differential equation with a hyperpolarization injection rate constant k_{W} and a relaxation rate constant of the build-up $k_{\mathrm{R}}^{\mathrm{bup}}$

$$\frac{\mathrm{d}P}{\mathrm{d}t} = (A-P)k_{\mathrm{W}} - k_{\mathrm{R}}^{\mathrm{bup}}P \tag{S6}$$

with A describing the theoretical maximum of hyperpolarization achievable, i.e., the thermal electron polarization in DNP. The solution of Eq. (S6) is a mono-exponential curve which can be compared with the phenomenological description of the build-up curve by $P(t) = P_0(1-e^{-t/\tau_{\mathrm{bup}}})$ to express the experimental parameters in terms of model parameters. Here, P_0 is the steady-state polarization and τ_{bup} the build-up time.

$$\tau_{\mathrm{bup}}^{-1} = k_{\mathrm{W}} + k_{\mathrm{R}}^{\mathrm{bup}} \tag{S7a}$$

$$P_0 = \frac{Ak_{\mathrm{W}}}{k_W + k_{\mathrm{R}}^{\mathrm{bup}}} = Ak_{\mathrm{W}}\tau_{\mathrm{bup}} \tag{S7b}$$

For the decay, k_{W} would be set to zero (MW off), leading to $\tau_{\mathrm{decay}}^{-1} = k_{\mathrm{R}}^{\mathrm{decay}}$.

Extending the one-compartment model to two uncoupled compartments with separate injection and relaxation rates is straightforward. Such a situation might be realized for a material consisting of two phases with different compositions (radical concentration, NMR-active spin concentration) such that each compartment follows its own mono-exponential

build-up (cf. Eqs. (S6), (S7)). Crucially, spin diffusion between the two compartments needs to be suppressed, e.g., through a resonance frequency difference rendering inter-compartment nuclear flip-flops energy non-conserving. If the frequency difference between the two compartments is small compared to the NMR linewidth such that the two compartments cannot be clearly discriminated through different peaks, the total measured signal describes the total magnetization created in the two compartments. In such a case, the resulting build-up would take a bi-exponential form

$$\begin{aligned} P &= P_{0,2}\left[\alpha\left(1-e^{-t/\tau_1}\right)+(1-\alpha)\left(1-e^{-t/\tau_2}\right)\right] \\ &= P_{0,2}\left[1-\alpha e^{-t/\tau_1}-(1-\alpha)e^{-t/\tau_2}\right] \end{aligned} \tag{S8}$$

with the relative weight of the two time constants α. Experimentally, four parameters are extracted from the build-up while in the theoretical model five parameters are required: two injection and relaxation rates each giving rise to the two steady-state polarizations and build-up times as well as the relative size of the compartments. Hence, for two uncoupled compartments, it is difficult to extract information about the individual compartments based on the above compartment model ansatz.

However, for infinitely many uncoupled compartments with some additional assumptions, we can describe stretched exponential build-ups: It was recently proposed[19] that for systems without spin diffusion and nuclear relaxation only through paramagnetic relaxation, the build-up can be described by a stretched exponential. In such a case the DNP transfer rate per lattice site and the relaxation scale with r^{-6}, with r being the distance between the nuclear spin and the electron as both processes are mediated by the hyperfine coupling between the paramagnetic center and the nucleus under consideration. This case can be considered as infinitely many uncoupled compartment (with only paramagnetic relaxation). Thus, our DNP injection rate has the same spatial scaling as the DNP transfer rate and

the paramagnetic relaxation. We write for this case $k_{W1} = \frac{k_{W0}}{r^6}$ and $k_{\mathrm{R}} = \frac{k_{R0}}{r^6}$, ignoring any angular dependence of the hyperfine interaction. To describe the polarization build-up of the total system, we can divide it into systems with the same distance to the paramagnetic center, treat these as single compartments and average over all subsystems. For this we find

$$\begin{aligned} P &= \frac{Ak_{W0}}{k_{R0}+k_{W0}}\frac{4}{3}\pi \int_{r_0}^{r_c} \mathrm{d}r\; r^3 \left(1 - e^{-\frac{k_{R0}+k_{W0}}{r^6}t}\right) \\ &= \frac{Ak_{W0}}{k_{R0}+k_{W0}}\frac{4}{3}\pi\frac{1}{4}\left[r^4 - \frac{2}{3}\left(\left(k_{R0}+k_{W0}\right)t\right)^{2/3}\Gamma\left(-\frac{2}{3}, \frac{k_{R0}+k_{W0}}{r_c^6}t\right)\right]_{r_0}^{r_b} + C \end{aligned} \tag{S9}$$

with Γ being the incomplete Gamma function. r_0 and r_c are a lower and upper cut-off radius around a paramagnetic center. The lower bound describes the spins that are invisible to NMR owing to their large hyperfine shift (quenched spins) and r_C would describe the Wigner-Seitz radius. C is the constant of integration and chosen as zero in the following. Since k_{W0}, k_{R0}, r_0, r_c and t being positive real numbers, this solution is valid. Since our polarization is positive and our time is a positive real number, we pick the real part of the lower incomplete Gamma function as our solution for the polarization build-up. We choose $r_0 = 0$ as the number of quenched spins is assumed to small compared to the total number of spins ($r_0 << r_C$). Furthermore, we choose $r_c = 1$ as this ensures that $P \in [0, 1]$. This can be interpreted as r_c defining the characteristic length scale of the system. The resulting build-up curve is fitted with a stretched exponential of the form

$$P = P_{0,s}\left(1 - e^{-(t/\tau_s)^{\kappa}}\right) \tag{S10}$$

with $P_{0,s}$ being the steady-state polarization of the stretched exponential build-up, τ_s the build-up time constant and κ the stretch exponent. Simulated build-ups of equation (S9) for $r_c = 1$ can be accurately fitted with a stretched exponential (Eq. (S10)) for a large range of $k_{W0}+k_{R0}$ with an example for this given in Fig. S17a. In Fig. S17b and c, the dependence of the stretched exponential build-up parameters based on $k_{W0} + k_{R0}$ is shown. The stretched

exponential build-up time $\tau_s \approx 3.229\,(k_{W0}+k_{R0})^{-1}$ as extracted from the fit in Fig. S17b.

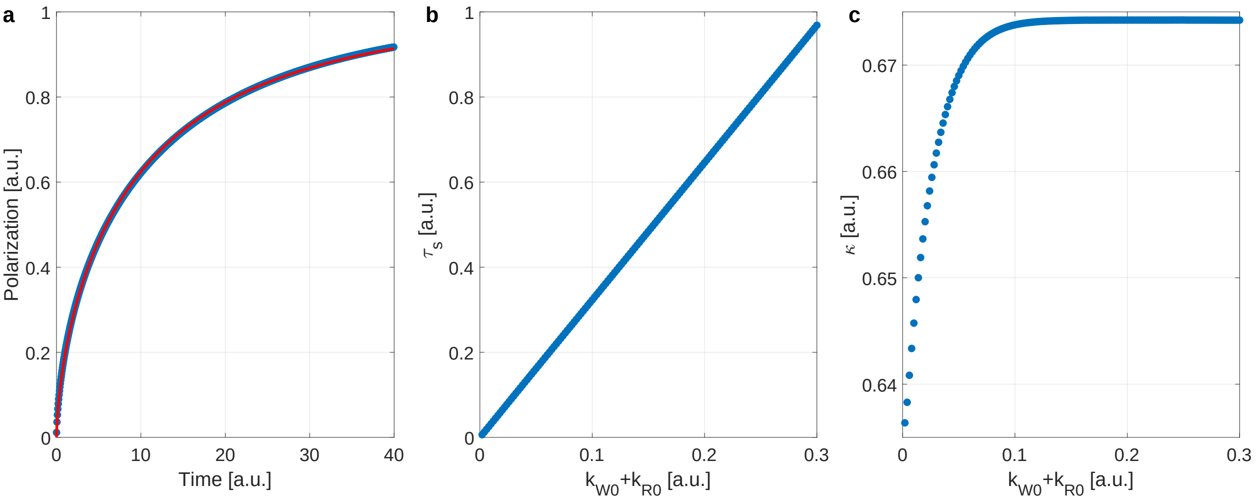

Figure S17: Numerical evaluation of Eq. (S9) ($r_c = 1$ and without the $P_{0,s} = \frac{Ak_{W0}}{k_{R0}+k_{W0}}\frac{4}{3}\pi\frac{1}{4}$ prefactor) and fitting the resulting curve with a stretched exponential. **a** A typical fit build-up and its stretched exponential build-up fit. Varying $k_{R0}+k_{W0}$ changes the characteristic time constant τ_S (**b**) and stretch exponent κ of a stretched exponential as defined in equation (S10). For the linear fit of τ_s a slope of 3.229 ± 0.001 is found.

The corresponding polarization decay/ relaxation after stopping the MW irradiation is straightforwardly derived from Eq. (S9) through replacing the $\left(1 - e^{-\frac{k_{R0}+k_{W0}}{r^6}t}\right)$ with $e^{-\frac{k_{R0}}{r^6}t}$ in the integral. This cancels the r_c^4 term in the solution. The corresponding experimental model would be $P = P_{0,s}e^{-(t/\tau_s)^\kappa}$.

All the experimentally measured exponents reported in Ref.[19] exponents are between 0.69 and 0.74. These values are slightly larger than the exponents shown in Fig. S17c, although they are within the uncertainty limits of the experimentally measured exponents. If a systematic difference between the measured and simulated exponents exists, two possible explanations could be the presence of weak spin diffusion in the experiment or the assumption of an angle-independent hyperfine coupling. It would be interesting to investigate if a weak spin diffusion would lead to a larger exponent, eventually reaching $\kappa = 1$ if spin diffusion in the system is significant. If this would be the case, the discrepancy between the simulated (cf. Fig. S17c) and an eventually measured exponent would indicate the balance between direct and spin diffusion mediated hyperpolarization transfer.

References

(1) von Witte, G.; Himmler, A.; Kozerke, S.; Ernst, M. Relaxation enhancement by microwave irradiation may limit dynamic nuclear polarization. *Physical Chemistry Chemical Physics* **2024**, *26*, 9578–9585.

(2) Bussandri, S.; Shimon, D.; Equbal, A.; Ren, Y.; Takahashi, S.; Ramanathan, C.; Han, S. P1 Center Electron Spin Clusters Are Prevalent in Type Ib Diamonds. *Journal of the American Chemical Society* **2024**, *146*, 5088–5099, Publisher: American Chemical Society.

(3) Nir-Arad, O.; Shlomi, D. H.; Manukovsky, N.; Laster, E.; Kaminker, I. Nitrogen Substitutions Aggregation and Clustering in Diamonds as Revealed by High-Field Electron Paramagnetic Resonance. *Journal of the American Chemical Society* **2024**, *146*, 5100–5107, Publisher: American Chemical Society.

(4) Palani, R. S.; Mardini, M.; Quan, Y.; Ouyang, Y.; Mishra, A.; Griffin, R. G. Dynamic Nuclear Polarization with P1 Centers in Diamond. *The Journal of Physical Chemistry Letters* **2024**, 11504–11509, Publisher: American Chemical Society.

(5) Himmler, A.; Albannay, M. M.; Von Witte, G.; Kozerke, S.; Ernst, M. Electroplated waveguides to enhance DNP and EPR spectra of silicon and diamond particles. *Magnetic Resonance* **2022**, *3*, 203–209.

(6) Terblanche, C. J.; Reynhardt, E. C. Room-temperature field dependence of the electron spin–lattice relaxation times of paramagnetic P1 and P2 centers in diamond. *Chemical Physics Letters* **2000**, *322*, 273–279.

(7) Terblanche, C. J.; Reynhardt, E. C.; van Wyk, J. A. 13C Spin–Lattice Relaxation in Natural Diamond: Zeeman Relaxation at 4.7 T and 300 K Due to Fixed Paramagnetic Nitrogen Defects. *Solid State Nuclear Magnetic Resonance* **2001**, *20*, 1–22.

(8) von Witte, G.; Kozerke, S.; Ernst, M. Two-electron two-nucleus effective Hamiltonian and the spin diffusion barrier. *Science Advances* **2025**, *11*, eadr7168.

(9) Rej, E.; Gaebel, T.; Boele, T.; Waddington, D. E.; Reilly, D. J. Hyperpolarized nanodiamond with long spin-relaxation times. *Nature Communications* **2015**, *6*, 8459, Publisher: Nature Publishing Group _eprint: 1502.06214.

(10) Waddington, D. E.; Boele, T.; Rej, E.; McCamey, D. R.; King, N. J.; Gaebel, T.; Reilly, D. J. Phase-Encoded Hyperpolarized Nanodiamond for Magnetic Resonance Imaging. *Scientific Reports* **2019**, *9*, 1–10, Publisher: Springer US _eprint: 1709.01851.

(11) Boele, T.; Waddington, D. E. J.; Gaebel, T.; Rej, E.; Hasija, A.; Brown, L. J.; McCamey, D. R.; Reilly, D. J. Tailored nanodiamonds for hyperpolarized $^{13}\mathrm{C}$ MRI. *Physical Review B* **2020**, *101*, 155416, Publisher: American Physical Society.

(12) Stoll, S.; Schweiger, A. EasySpin, a comprehensive software package for spectral simulation and analysis in EPR. *Journal of Magnetic Resonance* **2006**, *178*, 42–55.

(13) Reynhardt, E. C.; High, G. L.; van Wyk, J. A. Temperature dependence of spin-spin and spin-lattice relaxation times of paramagnetic nitrogen defects in diamond. *The Journal of Chemical Physics* **1998**, *109*, 8471–8477.

(14) Loubser, J. H.; Van Wyk, J. A. Electron spin resonance in the study of diamond. *Reports on Progress in Physics* **1978**, *41*, 1201–1248.

(15) Reynhardt, E. Spin lattice relaxation of spin-$\frac{1}{2}$ nuclei in solids containing diluted paramagnetic impurity centers. I. Zeeman polarization of nuclear spin system. *Concepts in Magnetic Resonance Part A* **2003**, *19A*, 20–35.

(16) Loubser, J. H. N.; Wright, A. C. J. A singly ionized N-C-N centre in diamond. *Journal of Physics D: Applied Physics* **1973**, *6*, 1129.

(17) van Wyk, J. A.; Loubser, J. H. N.; Newton, M. E.; Baker, J. M. ENDOR and high-temperature EPR of the N3 centre in natural type Ib diamonds. *Journal of Physics: Condensed Matter* **1992**, *4*, 2651.

(18) von Witte, G.; Ernst, M.; Kozerke, S. Modelling and correcting the impact of RF pulses for continuous monitoring of hyperpolarized NMR. *Magnetic Resonance* **2023**, *4*, 175–186.

(19) Jardón-Álvarez, D.; Reuveni, G.; Harchol, A.; Leskes, M. Enabling Natural Abundance 17O Solid-State NMR by Direct Polarization from Paramagnetic Metal Ions. *Journal of Physical Chemistry Letters* **2020**, *11*, 5439–5445.
